# Adaptation and Cryptic Pseudogenization in Penguin Toll-Like Receptors

**DOI:** 10.1093/molbev/msab354

**Published:** 2021-12-13

**Authors:** Steven R Fiddaman, Michal Vinkler, Simon G Spiro, Hila Levy, Christopher A Emerling, Amy C Boyd, Evangelos A Dimopoulos, Juliana A Vianna, Theresa L Cole, Hailin Pan, Miaoquan Fang, Guojie Zhang, Tom Hart, Laurent A F Frantz, Adrian L Smith

**Affiliations:** Department of Zoology, University of Oxford, Oxford, United Kingdom; Department of Zoology, Faculty of Science, Charles University, Prague, Czech Republic; Wildlife Health Services, Zoological Society of London, London, United Kingdom; Department of Zoology, University of Oxford, Oxford, United Kingdom; Biology Department, Reedley College, Reedley, CA, USA; Jenner Institute, University of Oxford, Oxford, United Kingdom; The Palaeogenomics and Bio-Archaeology Research Network, Research Laboratory for Archaeology and History of Art, University of Oxford, Oxford, United Kingdom; Departamento de Ecosistemas y Medio Ambiente, Facultad de Agronomía e Ingeniería Forestal, Pontificia Universidad Católica de Chile, Macul, Santiago, Chile; Section for Ecology and Evolution, Department of Biology, University of Copenhagen, Copenhagen, Denmark; BGI-Shenzhen, Beishan Industrial Zone, Yantian District, Shenzhen, China; BGI-Shenzhen, Beishan Industrial Zone, Yantian District, Shenzhen, China; Section for Ecology and Evolution, Department of Biology, University of Copenhagen, Copenhagen, Denmark; BGI-Shenzhen, Beishan Industrial Zone, Yantian District, Shenzhen, China; State Key Laboratory of Genetic Resources and Evolution, Kunming Institute of Zoology, Chinese Academy of Sciences, Kunming, China; Center for Excellence in Animal Evolution and Genetics, Chinese Academy of Sciences, Kunming, China; Department of Zoology, University of Oxford, Oxford, United Kingdom; School of Biological and Chemical Sciences, Fogg Building, Queen Mary University of London, London, United Kingdom; Institute of Palaeoanatomy, Domestication Research and the History of Veterinary Medicine, Faculty of Veterinary Medicine, Ludwig Maximilian University of Munich, Munich,Germany; Department of Zoology, University of Oxford, Oxford, United Kingdom

**Keywords:** Toll-like receptors, immunogenetics, pseudogenization, host–pathogen interaction, wildlife disease, avian immunology

## Abstract

Penguins (Sphenisciformes) are an iconic order of flightless, diving seabirds distributed across a large latitudinal range in the Southern Hemisphere. The extensive area over which penguins are endemic is likely to have fostered variation in pathogen pressure, which in turn will have imposed differential selective pressures on the penguin immune system. At the front line of pathogen detection and response, the Toll-like receptors (TLRs) provide insight into host evolution in the face of microbial challenge. TLRs respond to conserved pathogen-associated molecular patterns and are frequently found to be under positive selection, despite retaining specificity for defined agonist classes. We undertook a comparative immunogenetics analysis of TLRs for all penguin species and found evidence of adaptive evolution that was largely restricted to the cell surface-expressed TLRs, with evidence of positive selection at, or near, key agonist-binding sites in *TLR1B*, *TLR4*, and *TLR5*. Intriguingly, *TLR15*, which is activated by fungal products, appeared to have been pseudogenized multiple times in the *Eudyptes* spp., but a full-length form was present as a rare haplotype at the population level. However, in vitro analysis revealed that even the full-length form of *Eudyptes* TLR15 was nonfunctional, indicating an ancestral cryptic pseudogenization prior to its eventual disruption multiple times in the *Eudyptes* lineage. This unusual pseudogenization event could provide an insight into immune adaptation to fungal pathogens such as *Aspergillus*, which is responsible for significant mortality in wild and captive bird populations.

## Introduction

Penguins (Sphenisciformes) are a unique order of flightless, wing-propelled diving seabirds widely distributed across diverse coastal and island environments across the Southern Hemisphere. Between 18 and 20 extant species of penguin have been documented, represented within six well-defined genera (*Aptenodytes*, *Pygoscelis*, *Eudyptula*, *Spheniscus*, *Eudyptes*, and *Megadyptes*), with some debate surrounding the taxonomy (species/subspecies status) of certain recently diverged lineages (Frugone et al. 2018; Clements et al. 2019; Cole et al. 2019a, 2019b; Frugone et al. 2019; Pan et al. 2019; Vianna et al. 2020).

The recent release of high-coverage genomes from all extant penguin species (Pan et al. 2019) as part of the wider “Bird 10k” genomes project (Zhang et al. 2015) provides a unique opportunity to address comparative evolutionary questions across an entire avian order. One such comparison is to assess how the penguin immune system has adapted to the many, varied pathogen environments in which penguins are endemic. There is evidence that pathogen species richness changes as a product of biotic and abiotic factors, which are known to vary significantly across the ranges of penguins (Cumming and Guegan 2006; Kamiya et al. 2014; Poulin 2014; Uhart et al. 2020). It is likely, therefore, that a key part of penguin species radiation has been adaptation to the novel pathogens they encounter.

One body of evidence for pathogen-driven immunogenetic selection in wild animals comes from the major histocompatibility complex (MHC; Dionne et al. 2007; Sallaberry-Pincheira et al. 2016; O’Connor et al. 2020), which is important for presenting antigens to the adaptive immune system. However, investigating functional genetic diversity in the MHC is challenging in non-model organisms because of the complexity associated with extensive gene duplication and gene loss events (Cummings et al. 2010). Instead, other immune genes are increasingly being recognized as targets for pathogen-mediated selection, particularly those encoding pattern-recognition receptors of the innate immune system. One such family is the Toll-like receptors (TLRs), which are cell surface and endosomal receptors responsible for recognizing pathogen-associated molecular patterns (PAMPs) and initiating intracellular signaling cascades (Takeda et al. 2003). TLRs are functionally conserved across vertebrates (Roach et al. 2005; Boyd et al. 2007; Brownlie and Allan 2011), but are frequently found to be under positive selection, making them attractive loci to study adaptation to different pathogen pressures (Areal et al. 2011; Grueber et al. 2014; Králová et al. 2018; Świderská et al. 2018; Velova et al. 2018; Levy et al. 2020).

Birds typically have ten TLRs, of which *TLR3*, *TLR4*, *TLR5*, and *TLR7* are clear one-to-one orthologs of mammalian TLRs (Boyd et al. 2007; Brownlie and Allan 2011). In birds, *TLR1* and *TLR2* are both present as two-copy tandem duplications. Recent evidence suggests that the *TLR1* duplication pre-dates the reptile-mammalian divergence, since *TLR1A* clusters with mammalian *TLR10* in phylogenetic analysis (Velova et al. 2018). The *TLR2* duplication is less well understood; some mammals also have a pseudogenized *TLR2* as a second copy (Roach et al. 2005), but it remains unclear whether this duplication occurred prior to the reptile-mammalian divergence or twice independently (Temperley et al. 2008; Huang et al. 2011; Velova et al. 2018). *TLR15* is generally considered to be an avian- and reptilian-specific TLR (Boyd et al. 2012). However *TLR15* has recently been identified in the Australian ghost shark (*Callorhinchus milii*; Voogdt et al. 2018), suggesting that the origin of the receptor predates the divergence of the Chondrichthyes fish and tetrapods. Finally, *TLR21* is found in fish, amphibians, reptiles, and birds (Keestra et al. 2010; Yeh et al. 2013) but is absent in mammals.

Having found evidence of functional adaptation in the Gentoo penguin (*Pygoscelis papua* ssp.) *TLR4* and *TLR5* (Levy et al. 2020), we undertook a comprehensive analysis of penguin TLRs to consider processes that may have shaped the evolution of TLR-mediated immunity across this entire order of vertebrates. Using genomes derived from all extant species of penguin (Li et al. 2014; Pan et al. 2019), we investigate patterns of adaptive evolution in penguin TLRs. In addition, we examine a highly unusual case of cryptic pseudogenization of *TLR15* in the *Eudyptes* (crested) penguins, which may have important implications for the susceptibility of penguins to fungal pathogens.

## Results

### TLR and Pseudogene Identification

BLAST (Altschul et al. 1990) was used to identify TLR sequences in assembled penguin genomes generated using Illumina short-read sequences (Li et al. 2014; Pan et al. 2019). The majority of penguin species possessed representatives of each of the ten avian TLRs (*TLR1A*, *TLR1B*, *TLR2A*, *TLR2B*, *TLR3*, *TLR4*, *TLR5*, *TLR7*, *TLR15*, and *TLR21*). Representatives of the TLR1/2 family were missing in several species, likely due to assembly issues resulting from almost identical regions of gene conversion that are difficult to resolve with short-read sequencing. *TLR3*, *TLR4*, *TLR5* and *TLR7*, and *TLR15* were identified in all penguin species. *TLR21* was absent or found only as a fragment in several penguin species, similar to other studies of avian TLRs (Alcaide and Edwards 2011; Velova et al. 2018), suggesting there may be technical difficulties assembling the genomic region comprising *TLR21* rather than true absence in these species (Shultz and Sackton 2019).

We identified a pseudogene for *TLR5* in the Snares Crested penguin (*Eudyptes robustus*), caused by a cytosine to thymine substitution at position 1180 which led to a premature stop codon. In contrast to a previous report on the African penguin (*Spheniscus demersus*; Dalton et al. 2016), we found no evidence of pseudogenization of *TLR7*, and the gene was present and intact in all other penguin species. As described in detail below, we found a pseudogenized copy of *TLR15* in all of the eight *Eudyptes* spp., and an independent heterozygous pseudogenization in the Chinstrap penguin (*Pygoscelis antarcticus*) *TLR15*. Pseudogenes were removed from subsequent diversity and selection analyses due to the propensity to accumulate mutations through drift following pseudogenization (Li et al. 1981).

### Patterns of Diversity in Penguin TLRs

**Fig. 1.**
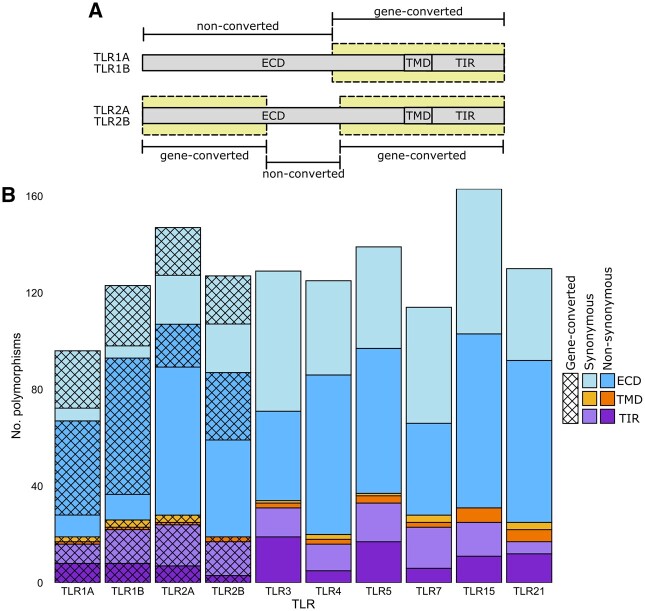
Diversity of penguin TLRs. (*A*) Schematic diagram of the regions in *TLR1A*/*1B* and *TLR2A*/*2B* which have undergone gene conversion in birds. Yellow boxes indicate regions of gene conversion. Abbreviations: ECD, extracellular domain; TMD, transmembrane domain; TIR, Toll/interleukin-1 receptor domain. (*B*) Number of polymorphisms per TLR (excluding pseudogenes), stratified according to domain and polymorphism type. Apparent polymorphisms in gene-converted regions of the TLR1/2 family are shown in hatched boxes as sequence differences between orthologs cannot be distinguished from differences between the gene-converted paralogs in genomes generated using exclusively short-read technology.

Alignments of penguin TLRs were analyzed for polymorphic sites. The TLR1/2 family in birds is known to have undergone multiple gene-conversion events (Huang et al. 2011; Velova et al. 2018; fig. 1*A*), which results in regions with almost exact sequence identity between the *TLR1A* and *TLR1B*, and *TLR2A* and *TLR2B* paralogs. Since all currently available high-coverage penguin genomes used short-read sequencing technology (Li et al. 2014; Pan et al. 2019), it remains difficult to confidently assign a read in a gene-converted region to the correct TLR paralog, which potentially inflates diversity statistics. We therefore excluded known gene-converted regions in the TLR1/2 family from diversity analysis. A total of 970 single nucleotide polymorphisms (SNPs) were identified in penguin TLRs, of which 550 (56.7%) were nonsynonymous changes (fig. 1*B*). In non-gene-converted TLRs, total polymorphism number was broadly similar, ranging from 114 (*TLR7*) to 163 (*TLR15*; fig. 1*B*). *TLR15* yielded the highest number of polymorphisms despite having comparatively few sequence representatives due to excluded pseudogenes in the *Eudyptes* spp. and the Chinstrap penguin (*P.antarcticus*).

### Positive Selection in Penguin TLRs

To investigate whether penguin TLRs have experienced diversifying selection, which could be an indicator of adaptation to different pathogen pressures, we fitted different site models (M1a/M2a and M7/M8) to multispecies sequence alignments using the *codeml* package in Phylogenetic Analysis by Maximum Likelihood (PAML) v. 4.9 (Yang 1997, 2007). All pseudogenes were removed prior to analysis, and only non-gene-converted regions of TLR1/2 family members were analyzed. In all cases, the M1a/M2a and M7/M8 results were comparable, so only the more conservative M1a/M2a *P*-values are reported in the following section (see supplementary table S1, Supplementary Material online, for all results). The cell surface TLRs *TLR1B*, *TLR4*, *TLR5*, and *TLR15* were found to evolve under positive selection in penguins (likelihood-ratio test; *P *=* *9.2 × 10^−4^, *P* = 9.1 × 10^−4^, *P* = 4.8 × 10^−6^, *P* = 0.023, respectively; supplementary table S1, Supplementary Material online). For the intracellular TLRs (*TLR3*, *TLR7*, and *TLR21*), positive selection was only supported for *TLR7* (*P *=* *0.13, *P* = 0.028, and *P *=* *0.65, respectively; supplementary table S1, Supplementary Material online).

**Fig. 2.**
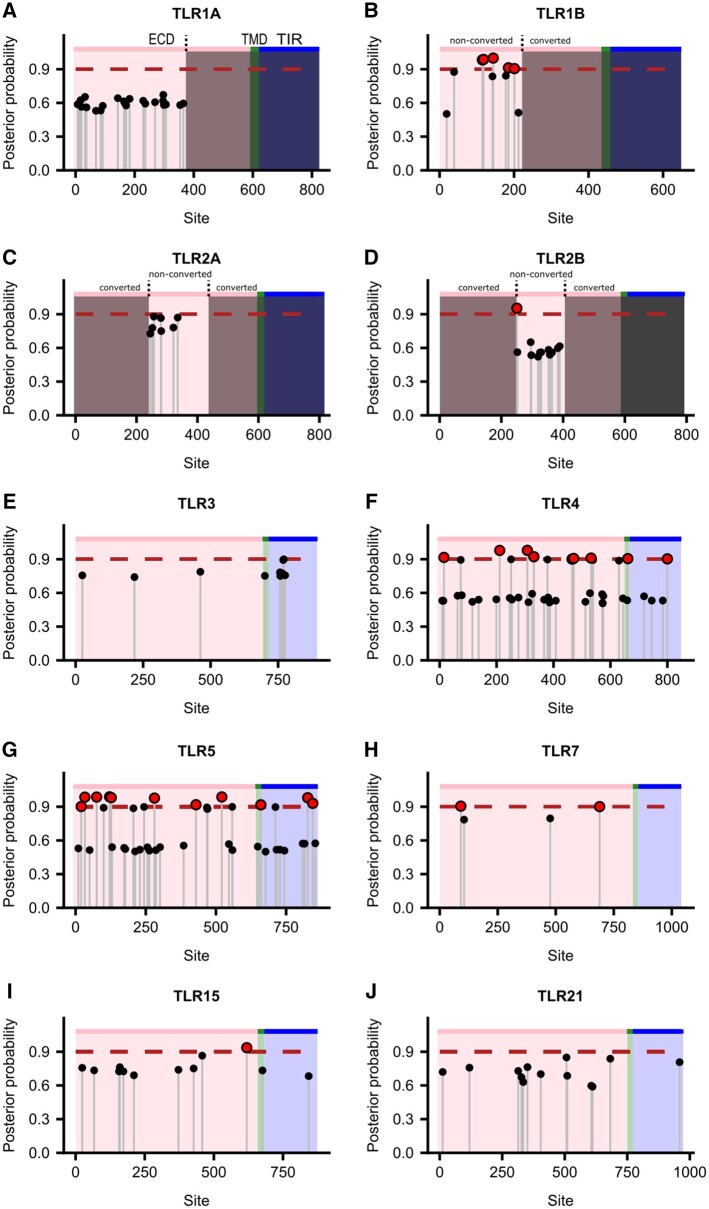
Locations of positively selected sites in penguin TLRs. Posterior probability of positively selected sites was determined using the Bayes Empirical Bayes procedure described in Yang et al. (2005). Red points indicate sites with posterior probability of selection of ≥0.90, whereas pink, green, and blue boxes denote the extracellular domain, transmembrane domain, and TIR domains of the TLR, respectively. Grayed-out regions in the TLR1/2 family denote excluded gene-converted regions.

**Fig. 3.**
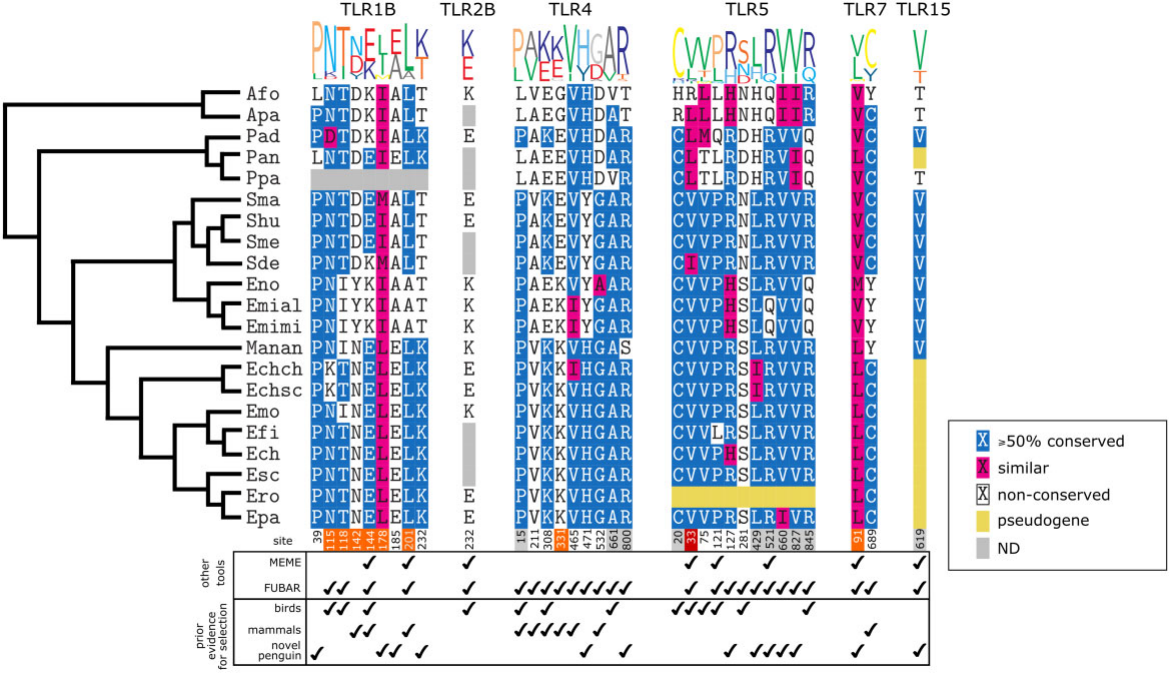
Alignments of positively selected sites. Amino acid sequence alignments of positively selected sites in all TLRs (excluding gene-converted regions of the TLR1/2 family). Sequence logo is colored according to the RasMol coloring convention, where amino acids of similar properties are grouped. Amino acids in the alignments are colored according to physicochemical conservation. Pseudogenes were excluded from the analysis, and are indicated by yellow boxes. Gray boxes in alignments indicate missing data. Site position (relative to the Afo coding sequence) is given below the alignment, and is colored according to whether the selected site is proximal to a site of known function (orange: the site is within 5 Å of a site of known function; red: the site has a known function; gray: distance from sites of known function could not be measured). For each site, “other tools” indicates whether a site was also found to be under positive selection by using the alternative methods of MEME and FUBAR, implemented in Datamonkey. Additionally, an indication is given under the alignment (“prior evidence for selection”) as to whether the site has previously been described as being positively selected in birds and/or mammals, or whether the site is a novel selected site in penguins (see supplementary table S2, Supplementary Material online for details). Sequence alignments and images were generated in the *msa* (v. 1.18.0) package in R (Bodenhofer et al. 2015) which implements the MUSCLE algorithm (Edgar 2004). Abbreviations: Afo, *Aptenodytes forsteri* (Emperor penguin); Apa, *Aptenodytes patagonicus* (King penguin); Pad, *Pygoscelis adeliae* (Adélie penguin); Pan, *Pygoscelis antarcticus* (Chinstrap penguin); Ppa, *Pygoscelis papua* (Gentoo penguin); Sma, *Spheniscus magellanicus* (Magellanic penguin); Shu, *Spheniscus humboldti* (Humboldt penguin); Sme, *Spheniscus mendiculus* (Galapagos penguin); Sde, *Spheniscus demersus* (African penguin); Eno, *Eudyptula novaehollandiae* (Fairy penguin); Emial, *Eudyptula minor albosignata* (White-flippered penguin); Emimi, *Eudyptula minor minor* (Little penguin); Manan, *Megadyptes antipodes antipodes* (Yellow-eyed penguin); Echch, *Eudyptes chrysolophus chrysolophus* (Macaroni penguin); Echsh, *Eudyptes chrysolophus schlegeli* (Royal penguin); Emo, *Eudyptes moseleyi* (Northern Rockhopper penguin); Efi, *Eudyptes filholi* (Eastern Rockhopper penguin); Ech, *Eudyptes chrysocome* (Southern Rockhopper penguin); Esc, *Eudyptes sclateri* (Erect-crested penguin); Ero, *Eudyptes robustus* (Snares Crested penguin); Epa *Eudyptes pachyrhynchus* (Fiordland penguin).

**Fig. 4.**
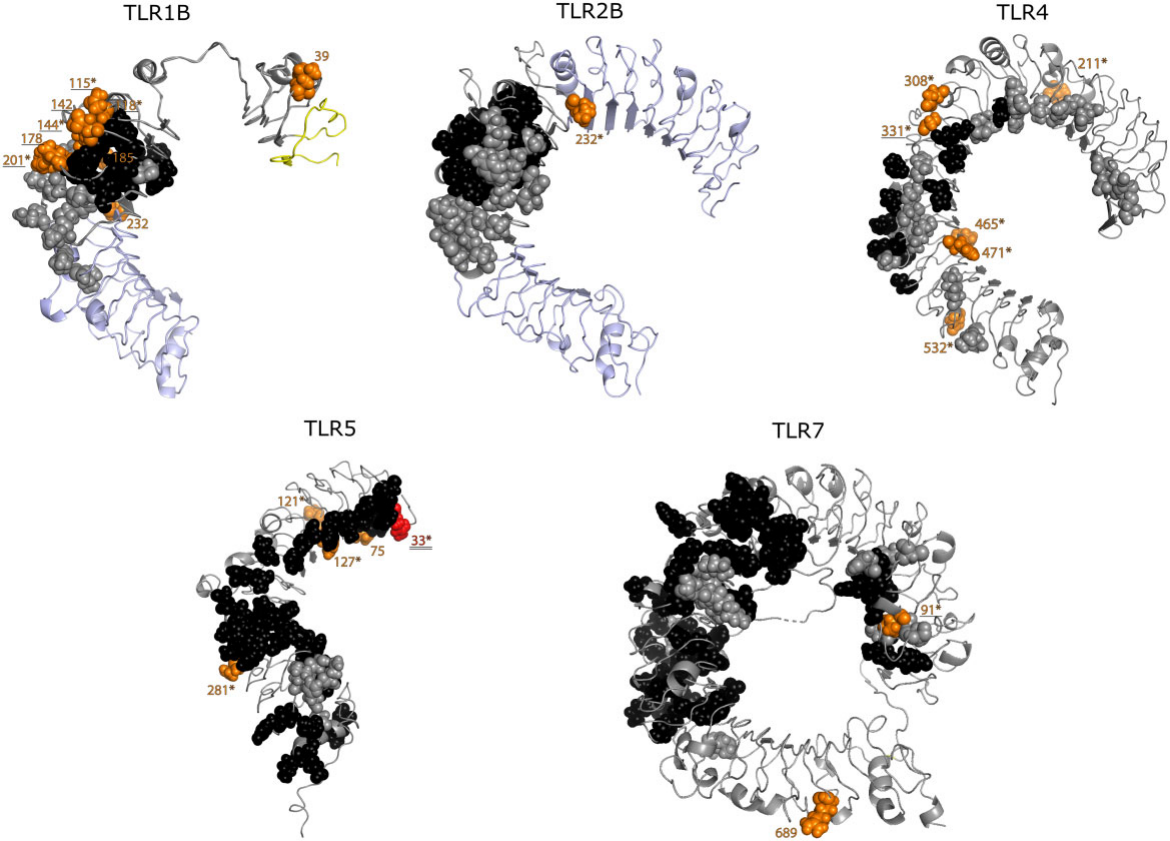
Locations of positively selected sites on penguin TLR ectodomains, relative to sites of known function. Sites that were determined to be under positive selection in PAML analysis were plotted on homology models of penguin TLRs (orange residues). Amino acids that are known to be agonist-binding sites are shaded black, whereas those involved in receptor dimerization are shaded gray. An underlined site indicates that it is proximal (<5Å) to a site of known function, and a site that is shaded red highlights a site of known function. An asterisk after the selected site position indicates that the site was determined to be positively selected by PAML and at least one other method (MEME or FUBAR). TLR backbones shaded blue indicate gene-converted regions in which positive selection could not be tested, and a yellow TLR backbone indicates a signal peptide. Certain sites are not displayed because they were omitted from the homology model template crystal structure (TLR4: sites 15, 661, and 800; TLR5: sites 20, 429, 521, 660, 827, and 845; TLR15: site 619). Distances to sites of known function could not be measured for these sites. High-resolution versions of these models can be found in supplementary figure S1, Supplementary Material online.

Using the Bayes Empirical Bayes procedure for inferring codon sites under selection implemented in model 2a of PAML (Yang et al. 2005), 33 positively selected sites were identified (posterior probability > 0.90) across the ten TLRs examined (figs. 2–4 andsupplementary table S2, Supplementary Material online). Of these, 28 were located in the extracellular (agonist-binding) domain, two were located in the transmembrane domain, and three were in the Toll/interleukin-1 receptor (TIR) homology domain (fig. 2).

To further validate the sites identified as being positively selected in PAML, we employed the Mixed Effects Model of Evolution (MEME; Murrell et al. 2012) and Fast Unconstrained Bayesian Approximation (FUBAR; Murrell et al. 2013) tests of positive selection. Sites identified by FUBAR largely agreed with those identified by PAML, and 26/33 sites were also significant for positive selection (posterior probability > 0.90; fig. 3 and supplementary table S3, Supplementary Material online). MEME analysis was not as consistent, and only 8/33 sites identified by PAML were corroborated in MEME (*P*-value ≤ 0.10; fig. 3 and supplementary table S3, Supplementary Material online), perhaps indicating that MEME is less powerful or more conservative than FUBAR.

Nine positively selected sites were identified in non-gene-converted regions of *TLR1B* (codons 39, 115, 118, 142, 144, 178, 185, 201, and 232; figs. 2–4) and a further codon was identified in *TLR2B* (232; figs. 2–4). Three of the sites in *TLR1B* have previously been identified as being positively selected in birds (115, 118, and 144; fig. 3; Velova et al. 2018), suggesting further diversification in the penguin lineage, and three of the sites have been identified in mammals (142, 144, and 201; fig. 3; Wlasiuk and Nachman 2010; Huang et al. 2011).

Interestingly, some of the sites identified in TLR1B are in close proximity to residues critical for TLR function. Residues 115, 118, 142, 144, and 178 of TLR1B are proximal (<5 Å) to sites involved in lipopeptide (Pam3CSK4) binding (Jin et al. 2007; Velova et al. 2018), suggesting there may have been changes in lipopeptide agonist recognition or specificity in penguins (fig. 4). Sites 142 and 201 are proximal (<5 Å) to known receptor dimerization sites (site 201 is adjacent to a known dimerization site) which could have functional implications for the formation of TLR heterodimers (Jin et al. 2007). Codons 43, 182, 189, and 237 (*TLR1B*) have not been previously identified as positively selected in birds or other taxa, so are diversified sites that are novel to penguins (fig. 3). Codon 232 (*TLR2B*) has previously been found to be positively selected in birds but is not proximal to a site of known function (fig. 3).

Almost all of the positively selected codons identified in penguin *TLR4* have been reported as positively selected in other taxa including mammals (codons 15, 211, 308, 331, 465, and 532; fig. 3; Nakajima et al. 2008; Vinkler et al. 2009; Wlasiuk and Nachman 2010; Areal et al. 2011; Shen et al. 2012; Fornůsková et al. 2013) and elsewhere in birds (codons 4, 308, and 661; fig. 3; Velova et al. 2018). Codons 471 and 800 have not been described as positively selected in other taxa, and therefore are uniquely diversified in the penguin lineage (fig. 3). In addition, we identified sites that could be functionally important due to their proximity to sites of known function. Site 308 is homologous to human site 298, adjacent to a site that has been implicated in lipopolysaccharide (LPS) hyporesponsiveness (Arbour et al. 2000; Ohto et al. 2012), and may be protective against malaria (Ferwerda et al. 2007). Site 331 is <5 Å from the LPS-binding site 314 (Park et al. 2009; Garate and Oostenbrink 2013; Paramo et al. 2013; Scior et al. 2013), so could be functionally important in the recognition of bacterial LPS. Taken together, these observations suggest that TLR4 has undergone ectodomain diversification in different lineages, which may be indicative of adaptation to changes in the profile or structure of agonists.

Several codons identified as positively selected in penguin *TLR5* have been identified elsewhere in birds (fig. 3; codons 20, 33, 75, 121, 281, and 845; Grueber et al. 2014; Vinkler et al. 2014; Velova et al. 2018). Furthermore, site 33 has previously been identified as a flagellin binding site (Yoon et al. 2012), which could imply a change of flagellin-binding preference. Several selected sites in TLR5 could not be modeled because no suitable crystal structure template covering the sites was available (sites 20, 429, 521, 660, 827, and 845) and distance from sites of known function could therefore not be determined. However, it is interesting to note that site 845 is one of two cosegregating sites that has recently been implicated in TLR5 signaling intensity differences in the Gentoo penguin species complex (*P.papua* ssp.; Levy et al. 2020), suggesting that this functional change is not restricted to the Gentoo penguin.

Two positively selected codons were identified in *TLR7*, one of which (689) is also selected in mammalian *TLR7* (Wlasiuk and Nachman 2010; Areal et al. 2011). Importantly, the other selected site (91) is adjacent to a known dimerization site of TLR7 (Zhang et al. 2016), which could have implications for TLR7 homodimer formation. We found one positively selected codon (619) in *TLR15* which has not been identified elsewhere in birds (figs. 2 and 3). Currently, insufficient data exist to determine whether this site is likely to be of functional significance, and the site could not be plotted on a homology model because a suitable crystal structure template is lacking.

### Pseudogenization of TLR15

**Fig. 5.**
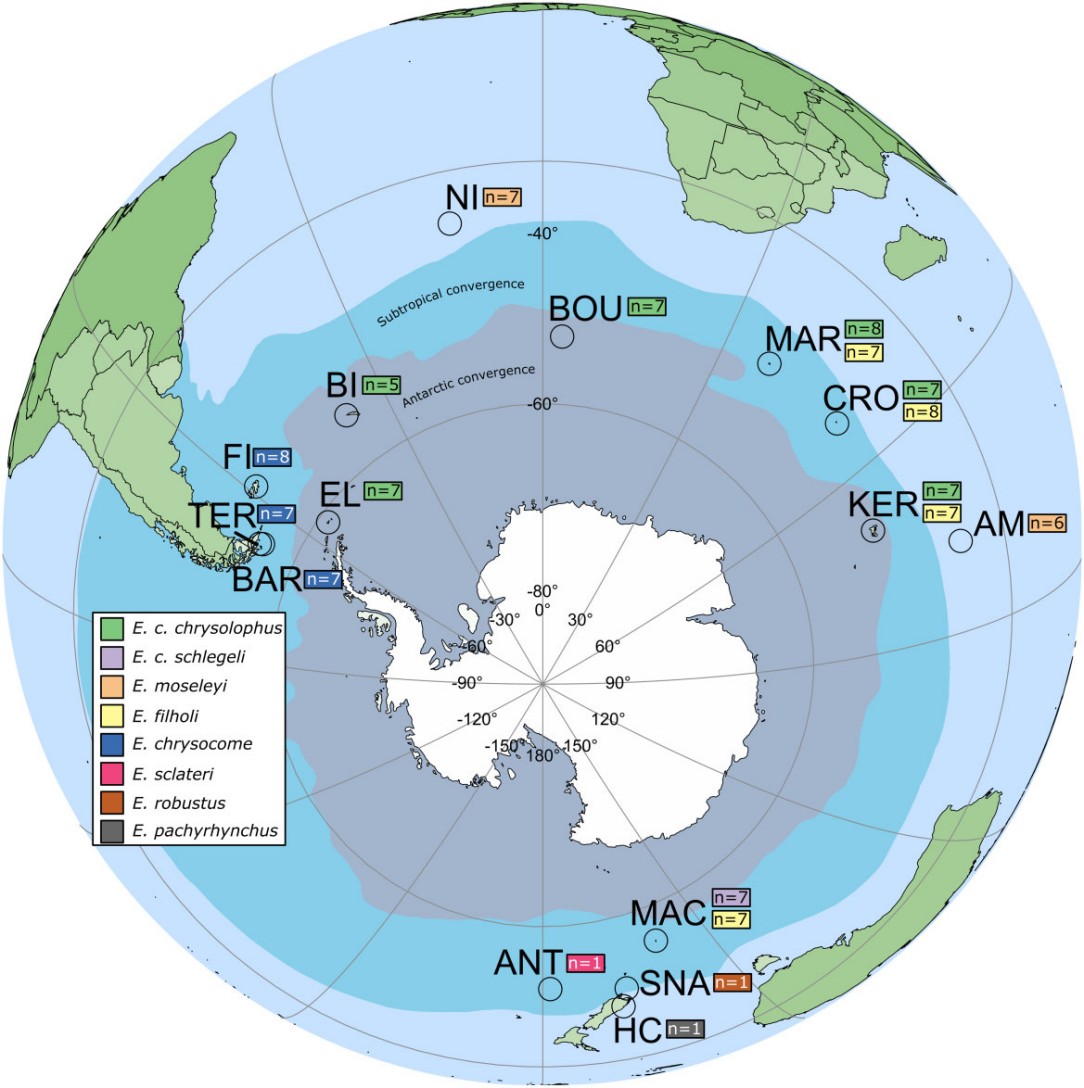
*TLR15* sequences used in pseudogene analysis by location and species. Number of individuals included in the analysis is given in boxes beside location labels, with color indicating the species. Location abbreviations: ANT, Antipodes Islands; AM, Amsterdam Island; BAR, Barnevelt Islands; BI, Bird Island, South Georgia; BOU, Bouvet Island; CRO, Crozet Islands; EL, Elephant Island, South Shetland Islands; FI, Falkland/Malvinas Islands; HC, Harrison Cove, New Zealand; KER, Kerguelen Islands; MAC, Macquarie Island; MAR, Marion Island; NI, Nightingale Island; SNA, The Snares, New Zealand; TER, Terhalten Island, Tierra del Fuego.

Analysis of *TLR15* sequences indicated that this gene has been disrupted (at least as a heterozygote) in seven of the eight reference genomes for the *Eudyptes* genus. The disrupting mutations (premature stop codon or indel introducing a frameshift) were often distinct in different species, suggesting several independent pseudogenization events. To explore this in more detail, the data set was supplemented with resequencing data from a larger number of individuals from five *Eudyptes* species/subspecies obtained from various locations around their natural ranges (fig. 5; *E. chrysolophus chrysolophus*, *n *=* *40; *E. chrysolophus schlegeli*, *n *=* *6; *E. moseleyi*, *n *=* *12; *E. filholi*, *n *=* *28; and *E. chrysocome*, *n *=* *21; total *n *=* *107; supplementary table S4, Supplementary Material online). *TLR15* sequences were extracted from these genomes, phased, and examined for the presence of pseudogene-causing mutations.

**Fig. 6.**
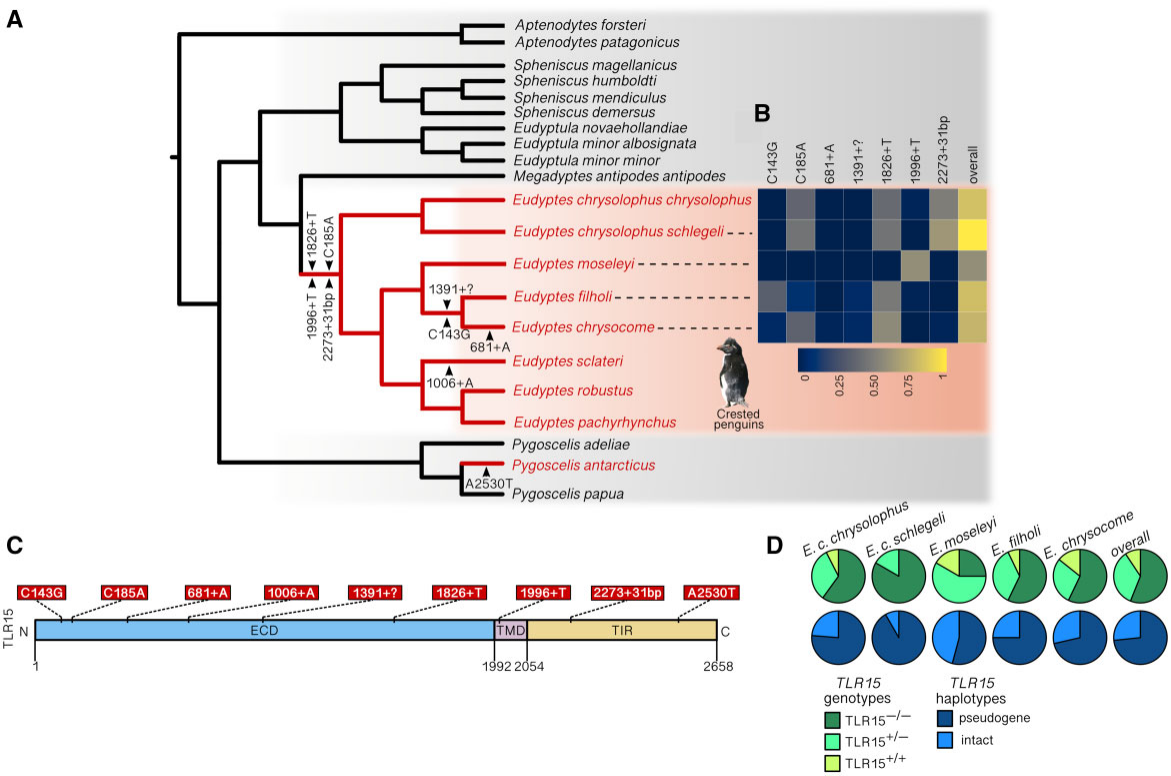
*TLR15* appears to have been pseudogenized repeatedly in *Eudyptes* penguins. (*A*) Cladogram of penguins indicating species where at least one individual was found to be at least heterozygous for a pseudogene-causing mutation in *TLR15* (red labels). Independent pseudogene events (as determined by at least one bird having only the specified pseudogene mutation) are indicated by black arrows at the latest branch at which the mutation could have occurred, given the taxonomic distribution. The topology of the phylogeny was taken from Pan et al. (2019) constructed with whole genome data sets where all nodes have bootstrap support of 100, apart from the split between the African penguin (*Spheniscus demersus*) and the Magellanic penguin (*Spheniscus magellanicus*), where bootstrap support = 97. (*B*) Heat map indicates the proportion of each of the sampled individuals that were at least heterozygous for the specified pseudogene-causing mutation in species for which population-level *TLR15* sequence data were available (supplementary table S8, Supplementary Material online). (*C*) Schematic diagram of *TLR15* shows the position of each independent pseudogene event relative to the gross architecture of the receptor (numbers are nucleotide positions). Six mutations were found in the extracellular domain (ECD), one in the transmembrane domain (TMD), and two in the TIR domain. “1391?” indicates an insertion that is too large to be resolved using short-read sequencing technology, but is likely to be in excess of ∼150 bp (and contains a stop codon). (*D*) *TLR15* haplotypes and genotypes of species for which population-level data were available. *TLR15*^−/−^ birds were homozygous for a pseudogene-causing mutation (or had one independent pseudogene in each copy); *TLR15^+/+^* birds had no evidence of a pseudogene-causing mutation; *TLR15^+/−^* birds had one heterozygous pseudogene event.

Most individuals had more than one mutation that, in isolation, could represent a pseudogene-causing event (i.e., more than one indel/premature stop codon). In order to identify independent pseudogenizations, we focused on individuals with only one disrupting mutation to exclude mutations arising subsequently by drift. Eight such events were identified in the *Eudyptes* spp., comprising two nonsense SNPs (C143G and C185A), four single nucleotide insertions (681+A, 1006+A, 1826+T, and 1996+T), and two larger insertions (2273+31 bp, and a large insertion at position 1391 of unknown length, denoted “1391+?”; fig. 6*A–C*). Although we were unable to determine whether the large insertion would have resulted in a frameshift, the first codon of this insertion is a stop codon, and thus yields a truncated sequence. In the case of all other indels, the reading frame of the gene was disrupted and the open reading frame (ORF) was terminated by a stop codon a short distance from the indel site. A further independent pseudogenization in *TLR15* was noted in the Chinstrap penguin, *P.antarcticus—*a heterozygous nonsense mutation (A2530T; fig. 6*A*). Since the mutation is towards the end of the TIR domain, it is unclear whether the protein would still retain functionality in this species, and it is not known how prevalent such mutations are in the absence of population-level data.

Based on their phylogenetic distribution, the *Eudyptes TLR15* pseudogenes were inferred to have different times of origin (fig. 6*A* and *B*). 681+A was only observed in the Southern rockhopper penguin (*E. chrysocome*; 1/42 haplotypes) and C143G and 1391+? were only observed in the Eastern and Southern rockhopper penguins (*E. filholi* and *E. chrysocome*; C143G: 18/56 haplotypes and 2/42 haplotypes, respectively; 1391+?: 3/56 haplotypes and 2/42 haplotypes, respectively). The 681+A, C143G and 1391+? mutations were therefore inferred to have arisen within the Rockhopper penguin lineage. C185A, 1826+T, 1996+T, and 2273 + 31 bp were found in multiple species and were therefore considered to be more ancient in origin. For instance, the 1826+T mutation was found in the Macaroni penguin (*E. c.chrysolophus*; 30/80 haplotypes), the Royal penguin (*E. c.schlegeli*; 5/12 haplotypes), the Eastern rockhopper penguin (*E. filholi*; 25/56 haplotypes), and the Southern rockhopper penguin (*E. chrysocome*; 22/42 haplotypes).

Analysis of phased haplotypes indicated that a majority of *Eudyptes* spp. individuals had evidence of pseudogenization in at least one copy of the gene. Frequency of pseudogenization ranged from 83.3% (Northern rockhopper penguin, *E. moseleyi*) to 100% of individuals (Royal penguin, *E. c. schlegeli*; fig. 6*B*) with a mean of 90.8% across all species. We found that 157/214 (73.36%) of haplotypes were pseudogenes, 60/107 (56.07%) of birds had pseudogenes for both copies (*TLR15*^*−/−*^), 37/107 (34.58%) were heterozygous for one pseudogene and one intact haplotype (*TLR15^+/^*^*−*^), and 10/107 (9.35%) were homozygous for an intact haplotype (*TLR15^+/+^*). At the species level, the prevalence of the *TLR15*^*−/−*^ genotype was broadly similar (50–60%; fig. 6*D*) in the best-sampled species (the Macaroni, Eastern rockhopper, and Southern rockhopper penguins; *E. c. chrysolophus*, *E. filholi*, and *E. chrysocome*, respectively) but varied more in the Royal penguin (*E. c. schlegeli*; 83.33%; *n *=* *6) and the Northern rockhopper penguin (*E. moseleyi*; 25.00%; *n *=* *12), which likely indicates variation due to under-sampling. In all species, *TLR15^+/+^* prevalence was low (0–16.67%; fig. 6*D*), indicating that individuals with intact genotypes are rare. Phasing also revealed an overwhelming preponderance of unique haplotypes (209 unique from a possible 214 derived from the 107 individuals included in the analysis), which may indicate a relaxation in selection pressure following pseudogenization and the propagation of mutations by drift.

### Functional Analysis of Full-Length TLR15

Having multiple independent, but non-fixed, pseudogenizations in a single genus would be highly unusual. We therefore sought to test the alternative, more parsimonious, hypothesis that the apparently intact *TLR15* in the *Eudyptes* was in fact a cryptic pseudogene that has subsequently acquired mutations that truncated the coding sequence. FLAG-tagged full-length TLR15 from *Aptenodytes forsteri* (Emperor penguin), *Fulmarus glacialis* (Northern fulmar), *Gallus gallus* (chicken), and a consensus *Eudyptes* intact TLR15 (which was itself a haplotype present in wild birds) were expressed in HEK-Blue Null1 NF-κB (nuclear factor kappa-light-chain-enhancer of activated B cells) reporter cells. Cells expressing these constructs were then challenged with a known TLR15 agonist—*Saccharomyces cerevisiae* (Brewer’s yeast) lysate (Boyd et al. 2012)—alongside the lysate of an important fungal pathogen in penguins, *Aspergillus fumigatus*.

**Fig. 7.**
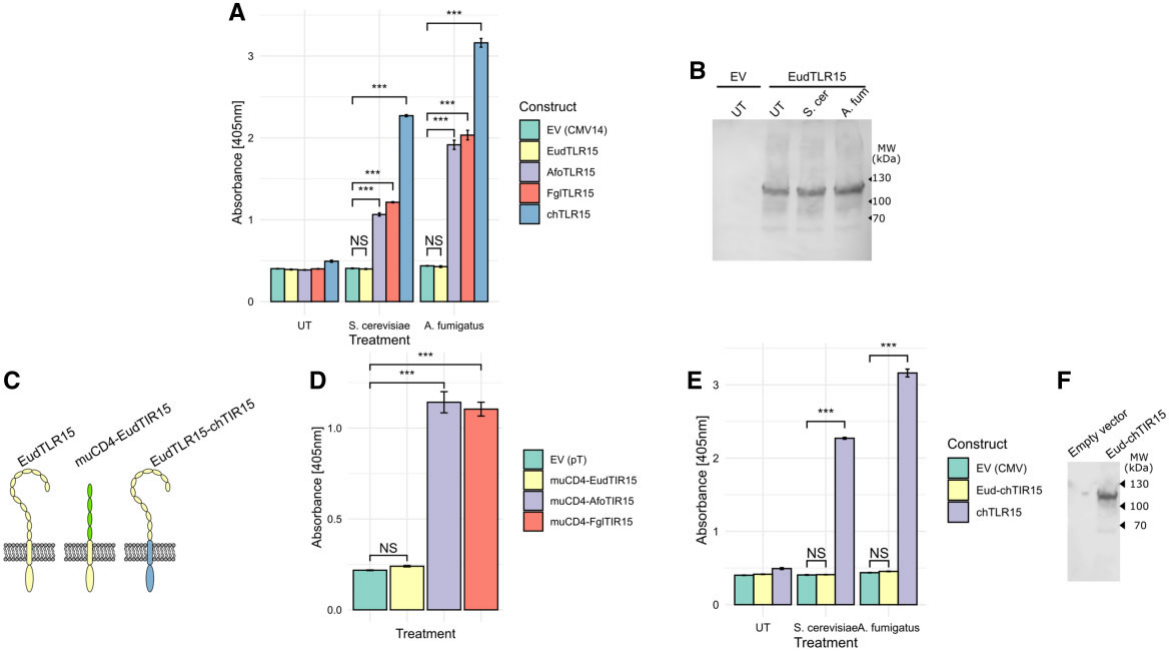
Full-length *Eudyptes* consensus TLR15 is nonfunctional, and is therefore a cryptic pseudogene. (*A*) Expression and stimulation of TLR15 from chicken (ch), Northern fulmar (Fgl), Emperor penguin (Afo), and a consensus intact Eudyptes TLR15 (Eud) in HEK-Blue Null1 NF-κB reporter cells. Cells expressing TLR15 constructs, or empty vector (EV) control, were challenged with brewer’s yeast (*S. cerevisiae*) or *A. fumigatus* lysates, or untreated (UT; medium control). (*B*) Immunoblot expression analysis of *Eudyptes* TLR15 in HEK-Blue Null1 NF-κB reporter cells with the same treatments as (*A*). (*C*) Schematic diagram of *Eudyptes* TLR15 chimaeras generated to functionally test the extracellular domain and intracellular domain independently. *Eudyptes* TLR15 TIR domain was attached to the extracellular domain from murine CD4 (muCD4-EudTIR15), and *Eudyptes* TLR15 extracellular domain was attached to the TIR domain from chicken TLR15 (EudTLR15-chTIR15). Equivalent chimaeric constructs were made for muCD4-AfoTIR15 and muCD4-FglTIR15. (*D*) NF-κB response to expression of muCD4-TIR domain chimaeras (and no additional stimulation). (*E*) NF-κB response to expression of Eud-chTIR15 or chTLR15 and stimulation with *S. cerevisiae* or *A. fumigatus* lysates. (*F*) Immunoblot expression analysis of EudTLR15-chTIR15 in HEK-Blue Null1 cells. CMV14 and pT (pTarget) refer to the expression vectors used for each construct. Statistical significance was considered to be *P *<* *0.05 and “***” denotes *P *<* *1 × 10^−7^ following Dunnett’s procedure for multiple comparisons; NS, not significant.

Interestingly, the Emperor penguin, Northern fulmar and chicken TLR15 all responded to both *S. cerevisiae* and *A. fumigatus* lysates, but neither could stimulate the activation of the *Eudyptes* TLR15 (fig. 7*A*), despite full-length protein clearly being expressed in the system (fig. 7*B*). This supports the hypothesis that even the seemingly intact *Eudyptes* TLR15 is nonfunctional, in contrast to other bird species. We then sought to identify the location of the lesion that gives rise to the nonfunctional phenotype by means of chimaeric TLR constructs (fig. 7*C*). To test for TIR domain functionality, a constitutively active form of the *Eudyptes* TLR15 transmembrane and TIR domain was constructed with the extracellular domain of murine CD4 (Medzhitov et al. 1997; fig. 7*C*). Similar constitutively active constructs were made for TLR15 from the Emperor penguin and the Northern fulmar as controls. The muCD4-EudTIR15 chimaera did not signal (whereas the control constructs did), suggesting a critical function-affecting lesion in the *Eudyptes* TLR15 TIR domain (fig. 7*D*). Finally, to test the extracellular domain of *Eudyptes* TLR15, a further chimaera was constructed by attaching the chicken TLR15 TIR domain (as a known-functional TIR domain) to the *Eudyptes* TLR15 extracellular domain. This construct was also nonresponsive to both *S. cerevisiae* and *A. fumigatus* lysates (fig. 7*E*), despite clearly being expressed (fig. 7*F*). Taken together, these results indicate that the seemingly intact *Eudyptes TLR15* is a cryptic pseudogene—one or more disabling (but not truncating) mutations rendered the receptor nonfunctional prior to its eventual overt pseudogenization by drift in subsequent lineages.

### Loss-of-Function Mutation

As the mechanism of action of TLR15 signaling has not been elucidated, we could not identify candidate sites for cryptic pseudogenization based on their proximity to sites of known function. Instead, we used a homology-based method to identify sites that are distinct in *Eudyptes* compared with other birds (supplementary tables S5–S7 and figs. S3, Supplementary Material online). Ten sites were identified that were distinct in *Eudyptes* compared with the rest of birds, and of these, three sites were highly conserved across a wider analysis of vertebrates (161, 736, and 787; supplementary table S5 and fig. S3, Supplementary Material online). Two polymorphisms were also found to be likely deleterious (L161P, extracellular domain, and L683S, Transmembrane/TIR domain; supplementary tables S6 and S7, Supplementary Material online) when applied to the chicken TLR15 sequence.

## Discussion

Immune system genes, particularly the TLRs, are frequently found to be hotspots of diversity and positive selection in birds (Alcaide and Edwards 2011; Grueber et al. 2014; Králová et al. 2018; Świderská et al. 2018; Velova et al. 2018; Shultz and Sackton 2019). This is unsurprising given the strong bilateral selection pressures at the host-pathogen interface. Penguins radiated from Australia/New Zealand in the early Miocene into highly diverse niches (Vianna et al. 2020), from the tropical Galápagos Islands through the temperate forests and rocky shores of southern hemisphere coastlines and islands, to the sea ice of Antarctica (Ropert-Coudert et al. 2014; Pan et al. 2019; Vianna et al. 2020). These diverse biotic and abiotic conditions are likely to foster distinct pathogen assemblages (Cumming and Guegan 2006; Kamiya et al. 2014; Poulin 2014; Uhart et al. 2020), which in turn has exerted a selection pressure on penguins to adapt to the different threats they pose. In this study, we found evidence of adaptive evolution at key functional sites in penguin TLRs, which strongly implies change of pathogen recognition function. In addition, we described a highly unusual cryptic pseudogenization event in the relatively understudied *TLR15* in the crested (*Eudyptes* spp.) penguins, which could have significant implications for how penguins respond to important pathogens such as *Aspergillus* spp.

### Penguin TLRs Have Undergone Diversifying Selection at Key Functional Sites

TLR orthologs retain specificity for particular agonists over large evolutionary distances (Roach et al. 2005). Despite this, TLRs in vertebrates frequently undergo diversifying selection through coevolution with pathogens (Areal et al. 2011; Velova et al. 2018; Liu et al. 2020). Even closely related taxa may show substantial variability in molecular phenotypes of individual TLRs (Králová et al. 2018; Levy et al. 2020; Těšický et al. 2020). Our findings are similar to those reported elsewhere in birds. Positive selection is more common in lipid-, glycan-, and protein-recognizing TLRs (TLR1B, TLR4, TLR5, and TLR15), compared with nucleic acid-recognizing TLRs as reported by others (Velova et al. 2018). Furthermore, the extracellular (LRR) domains of TLRs (which interact directly with PAMPs) were found to have many more positively selected sites than the intracellular (TIR) domains. An excess of positively selected sites in the extracellular domain of TLRs has been widely documented (Areal et al. 2011; Grueber et al. 2014; Świderská et al. 2018; Velova et al. 2018), likely due to the interaction with components of PAMPs that are themselves under selection to evade recognition (Andersen-Nissen et al. 2005). The TIR domain, on the other hand, interacts with host adaptor proteins and is typically under purifying selection (Mikami et al. 2012). The finding of an excess of positively selected sites in the extracellular domains of penguin TLRs could indicate adaptation to different pathogen pressures in different penguin species.

Further evidence of changing receptor function comes from several positively selected sites being at, or near, positions of known functional importance. For instance, residues 115, 118, 142, 144, and 178 of TLR1B, residues 308 and 331 of TLR4, and residue 33 of TLR5 are all at, or within 5 Å of, sites of known agonist-binding function (Arbour et al. 2000; Jin et al. 2007; Park et al. 2009; Ohto et al. 2012; Garate and Oostenbrink 2013; Paramo et al. 2013; Scior et al. 2013). All but one (TLR1B, site 178) of these sites have been found to be positively selected in mammals and/or birds in other studies (Nakajima et al. 2008; Vinkler et al. 2009; Wlasiuk and Nachman 2010; Areal et al. 2011; Huang et al. 2011; Shen et al. 2012; Fornůsková et al. 2013; Grueber et al. 2014; Velova et al. 2018), implying that these sites undergo recurrent positive selection to change function in different vertebrate lineages.

### Pseudogenization of TLR5 in the Snares Crested Penguin

While gathering sequences, we discovered a *TLR5* pseudogene in the Snares Crested penguin (*E.robustus*). *TLR5* pseudogenization has occurred multiple independent times in different bird species across several orders (Passeriformes, Psittaciformes, Cariamiformes, Trogoniformes, Phaethontiformes, Eurypygiformes, and Apodiformes; Alcaide and Edwards 2011; Bainova et al. 2014; Velova et al. 2018). The discovery of a *TLR5* pseudogene in the Snares Crested penguin represents the first description of a *TLR5* pseudogene in the Sphenisciformes. *TLR5* has also been lost independently from several other vertebrate lineages, including pigs, Yangtze river dolphin, pinnipeds, pangolins, tuatara, and clownfish (Liu et al. 2020; Sharma et al. 2020). The conditions under which *TLR5* pseudogenization occurs remain unclear, although it is interesting to note that *TLR5* pseudogenization invariably occurs at or near terminal branches in vertebrate phylogeny, suggesting a recent change in pathogen exposure. It is also interesting to note that the loss of *TLR5* has consequences in other vertebrates. For instance, the common TLR5-392^STOP^ variant in humans is associated with increased susceptibility to Legionnaires’ disease (Hawn et al. 2003). However, it is also plausible that the loss of *TLR5* is adaptive, akin to *TLR4*-null mice being resistant to endotoxic shock (Qureshi et al. 1999)

### Cryptic Pseudogenization in *Eudyptes* spp. TLR15

TLR15 is a comparatively understudied receptor, first described as being upregulated in the caecum of chickens infected with *Salmonella enterica* serovar Typhimurium (Higgs et al. 2006). Unlike most other TLRs, a canonical agonist has not been described for TLR15. There is evidence that TLR15 responds to the N-terminal diacylated lipopeptide of the hemagglutinin protein of *Mycoplasma synoviae* in a TLR1- and TLR2-independent manner (Oven et al. 2013). Another study identified a heat-labile and phenylmethylsulfonyl fluoride-abrogable yeast-derived agonist as being capable of activating TLR15 (Boyd et al. 2012), whereas a further report postulates that TLR15 is activated not by a conventional agonist, but rather by receptor cleavage induced by microbe-derived proteases (de Zoete et al. 2011). TLR15 faces the extracellular space (de Zoete et al. 2011) and is unusual for TLRs of this type for having a low rate of adaptive evolution (despite high levels of variation) in birds that is more akin to the intracellular nucleic-acid recognizing TLRs (*TLR3*, *TLR7*, and *TLR21*; Velova et al. 2018). This could be indicative of an unusual mechanism of stimulation, such as through cleavage by microbial proteases.

Initial examination of the penguin reference genomes suggested that *TLR15* had been pseudogenized multiple times in the *Eudyptes* spp., with a further independent pseudogenization in the Chinstrap penguin (*P.antarcticus*). Since several of the mutations were widespread in our population-level analysis of >100 *Eudyptes* penguins, we concluded that at least some of the disruptive events had taken place in the common ancestor to all *Eudyptes* penguins. It is worth considering whether this pattern may have arisen through modern introgression between lineages but this is unlikely. There is evidence for genome-wide introgression (up to 25%) between some specific pairings of *Eudyptes* penguins; for instance in Royal and Erect-crested penguins (*E. c.schlegeli* and *Eudyptes sclateri*, respectively; Vianna et al. 2020). However, with other *Eudyptes* spp., introgression is absent or minimal; for example between Royal and Eastern rockhopper penguins (*E. c.schlegeli* and *E. chrysocome filholi*, respectively) or between Macaroni and Eastern rockhopper penguins (*E. c.chrysolophus* and *E. c.filholi*, respectively; Vianna et al. 2020). Of note, the “basal” disrupted TLR15 haplotypes were shared across species irrespective of the levels of genome-wide introgression. For instance, the 1826+T frameshift mutation was found at high frequencies in divergent pairs of species with little evidence of introgression; 30/80 haplotypes in the Macaroni penguin (*E. c.chrysolophus*) and 25/56 haplotypes in the Eastern rockhopper penguin (*E. c.filholi*) implying acquisition by descent, rather than introgression. The fact that four independent pseudogenes were found to be basal to the genus is likely to have been a result of incomplete lineage sorting in the early evolution of the crested penguins. Furthermore, sequence intact haplotypes were present in the populations as part of heterozygote (*TLR15^+/^*^*−*^; 35.51%) or as homozygote (*TLR15*^+/+^; 9.35%) genotypes. Given that it would be highly unusual to have so many independent pseudogenizations of a gene in a single genus found alongside coding-sequence intact haplotypes, we elected to test whether the consensus intact genotype was functional or a cryptic pseudogene.

Although a canonical agonist has not been defined for TLR15, it is clear that this TLR is activated by yeast and fungal-derived products (de Zoete et al. 2011; Boyd et al. 2012). *Aspergillus* spp. are common fungal pathogens of penguins and other birds, both captive and wild (Khan et al. 1977; Obendorf and McColl 1980; Flach et al. 1990; Graczyk and Cockrem 1995; Hocken 2000; Beernaert et al. 2010). Given the importance of *Aspergillus* spp., we hypothesized that TLR15 may play a role in responding to *Aspergillus* spp. infection. It is clear that TLR15 from chicken, Northern fulmar, and Emperor penguin responded to *A.fumigatus* lysate, as well as a previously defined agonist source, brewer’s yeast (*S.cerevisiae*) lysate (Boyd et al. 2012). By contrast, the consensus intact *Eudyptes* spp. TLR15 was incapable of signaling in response to either of these agonists, despite being expressed in its full-length form. The failure of constitutively active forms of the *Eudyptes* TLR15 TIR domain or chimaeras (extracellular domain of *Eudyptes* TLR15 fused with the strongly signaling chicken TIR domain) to rescue activity suggests that there are multiple lesions across the TLR sequence (at least one in each domain). These findings suggest that receptor functionality was lost prior to the overt gene disruptions evident across the *Eudyptes*, and that overt pseudogenization was a result of relaxed purifying selection pressure on the (nonfunctional) gene.

It is therefore likely that the intact *Eudyptes* spp. *TLR15* haplotype represents a cryptic pseudogene, in the sense that the open reading frame is complete in the genome and is capable of yielding a full-length protein, but which lacks functionality. Cryptic pseudogenes are difficult to detect without functional analysis or an overt phenotype, and have only rarely been reported (Balakirev and Ayala 1996; Wolfe and dePamphilis 1998). More commonly, a gene that undergoes duplication may experience functional redundancy and subsequent loss of one copy through pseudogenization (Zhang 2003). However, a duplication/pseudogenization scenario does not fit with *Eudyptes TLR15*, since many of the truncating mutations were homozygous, we did not identify any triallelic sites, and the observed read depth of *TLR15* in reference genomes (Pan et al. 2019) was comparable to coding sequences across the rest of the genome (supplementary fig. S2, Supplementary Material online). It is also unlikely that *TLR15* experienced any functional redundancy with other TLRs, since *TLR15* is the sole family member and recognizes agonists that are distinct from other TLR families (Boyd et al. 2012). It is important to note that the radiation of *Eudyptes* penguins coincided with, and was perhaps driven by, a period of great flux with the emergence of sub-Antarctic and temperate islands in the Plio-Pleistocene (Cole et al. 2019b; Vianna et al. 2020). Small population dynamics associated with colonization of new islands may have fostered the survival and local fixation of a loss-of-function *TLR15* variant that subsequently drifted to fixation across the genus.

Although the mechanism of action of TLR15 has not been fully elucidated, it was clear that functionally disruptive changes exist in both the extracellular (leucine-rich repeat) domain and in the transmembrane/TIR domain. We identified a number of sites that differ in *Eudyptes* penguins that are otherwise well conserved across birds and other vertebrates (supplementary tables S5–S7 and fig. S3, Supplementary Material online). Using PROVEAN (Choi and Chan 2015) and SIFT (Ng and Henikoff 2003), we identified two sites that were predicted to be deleterious when the chicken TLR15 sequence was altered (L161P and L647S; site positions refer to the Emperor penguin sequence). Amino acid site 161 is located in the extracellular domain in an extended loop between LRR3 and LRR4, whereas site 647 is within the transmembrane domain. Additional work to functionally test the identified sites could give a better insight into the mechanism of TLR15 signaling, and into the origin of the cryptic pseudogene in *Eudyptes* spp.

The consequence of *TLR15* loss in penguins remains unclear. A simple prediction would be that birds with intact *TLR15* (e.g., the Emperor penguin) are more resistant to *A.fumigatus* infection compared with *Eudyptes* species. However, comparing incidence of aspergillosis in Emperor penguins and *Eudyptes* spp. is problematic because they are not sympatric in the wild, and are maintained at different climate conditions in captive settings (AZA Penguin Taxon Advisory Group 2014). There is evidence that several other penguin species are susceptible to aspergillosis, such as Gentoo penguins (genus: *Pygoscelis*; Flach et al. 1990), Magellanic penguins (genus: *Spheniscus*; Carrasco et al. 2001; Xavier et al. 2007; Krol et al. 2020), and Little penguins (genus: *Eudyptula*; Obendorf and McColl 1980; Hocken 2000). However, multiple other factors, such as environmental conditions and general health, are known to increase risk of mycoses (Beernaert et al. 2010), so elucidating the contribution of genetics to aspergillosis resistance is difficult. Future work to evaluate the functionality of *TLR15* in other penguin and bird species, followed by a prospective cohort study of aspergillosis infection in comparable penguin populations will greatly enhance our understanding of the genetic contribution to disease resistance. Penguins are popular attractions at zoos around the world, and the ability to identify genetically susceptible populations will help safeguard vulnerable animals. Similarly, climate change is predicted to introduce novel pathogens to wild populations, and so identifying genetically at-risk populations is valuable from a conservation perspective (Cohen et al. 2020).

### Conclusions

TLRs are under constant evolutionary pressure to adapt to a changing pathogen landscape. Penguins are an ideal taxon to study TLR adaptation because their extensive geographical range undoubtedly means that pathogen exposure is distinct between species and genera. In this study, we provided evidence of widespread adaptive evolution in TLRs across penguin phylogeny. Further, we recapitulated patterns of adaptive evolution of particular TLRs and functional sites that have been reported in other birds and vertebrate taxa. We also reported a highly unusual cryptic pseudogenization event in *Eudyptes TLR15*, which recognizes fungal products and may be involved in the recognition of pathogens such as *Aspergillus* spp. This gene evidently lost function in the common ancestor to extant *Eudyptes* and then accumulated multiple overt disruptive mutations, at least some of which occurred in basal lineages. Aside from contributing to our understanding of penguin susceptibility to aspergillosis, the cryptic pseudogenization of *TLR15* provides an insight into the process of trait erosion in wild animals and the processes involved in gene loss.

## Materials and Methods

### Sequence Retrieval

Twenty-one assembled penguin genome sequences were downloaded from the GigaScience Database (doi: http://gigadb.org/dataset/100649) from two studies exploring penguin evolution (Li et al. 2014; *A.forsteri* and *Pygoscelis adeliae*; Pan et al. 2019; all other penguin species). TLR sequences from the annotated Emperor penguin (*A.forsteri*) genome were used as query sequences (*TLR1A*: XM_009280175.1; *TLR1B*: XM_009280152.2; *TLR2A*: XM_009283352.2; *TLR2B*: XM_009283317.1; *TLR3*: XM_009277378.2; *TLR4*: XM_009282256.2; *TLR5*: XM_009275754.1; *TLR7*: XM_009278529.2; *TLR15*: XM_009288440.1; *TLR21*: XM_009282498.1). Data sets of predicted coding genes were queried using default sensitivity parameters in a local BLASTn search. Significant hits of *E *≈* *0 were retrieved. On occasions where significant hits were not retrieved from the predicted coding gene database, assembled genomes were queried using the genomic records from which the above *A. forsteri* mRNA sequences were derived. Finally, the collection of TLR sequences was supplemented by the available representatives from the Adélie penguin (*P. adeliae*; *TLR1A*: XM_009321398.1; *TLR1B*: XM_009332470.1; *TLR2A*: XM_009329794.1; *TLR2B*: XM_009329795.1; *TLR3*: XM_009330657.1; *TLR4*: XM_009319316.1; *TLR5*: XM_009333665.1; *TLR7*: XM_009318873.1; *TLR15*: XM_009319611.1) and with subclades of the Gentoo penguin (*P.papua* ssp.) from the Indian ocean and West Antarctic Peninsula (Levy et al. 2020; accessions: MN394307, MN394343, MN313097, MN313136, MN312948, and MN312985). Population-level data for *TLR15* in the *Eudyptes* penguins (*E. c.chrysolophus*, *n *=* *40; *E. c.schlegeli*, *n *=* *7; *E. chrysocome*, *n *=* *22; *E. filholi*, *n *=* *27; *E. moseleyi*, *n *=* *13) were obtained from whole-genome data generated in (Frugone et al. 2019; Vianna et al. 2020) and unpublished data.

### Alignments and Polymorphism Identification

Following the removal of introns (where appropriate) by reference to the annotated *A.forsteri* sequence, sequences were aligned using MUSCLE (v. 3.8.425; Edgar 2004) in Geneious Prime (2019.0.3). Sequences were truncated to the length of the coding sequence, with the start codon confirmed by the presence of a signal peptide in the amino acid sequence immediately downstream as determined by the Phobius web server (Kall et al. 2004, 2007). Polymorphisms in TLR sequences were identified using DnaSP v. 6.12 (Rozas et al. 2017).

### Positive Selection Analysis

To detect signatures of positive selection, multiple alignments were analyzed with the *codeml* program in PAML v. 4.9 (Yang 1997, 2007) with the F3X4 codon frequency model, using the phylogenetic tree from (Pan et al. 2019). Various models were fitted to the multiple alignments: M1a (neutral model; two site classes: 0 < *ω*_0_ < 1 and *ω*_1_ = 1); M2a (positive selection; three site classes: 0 < *ω*_0_ < 1, *ω*_1_ = 1 and *ω*_2_ > 1); M7 (neutral model; values of ω fit to a beta distribution where *ω*  >  1 is disallowed); M8 (positive selection; similar to M7 but with an additional codon class of ω  >  1). Likelihood ratio tests were performed on pairs of models to assess whether models allowing positively selected codons gave a significantly better fit to the data than neutral null hypothesis of neutral codon evolution could be rejected (*P* < 0.05), the posterior probabilities of codons under selection in M2a and M8 were inferred using the Bayes Empirical Bayes algorithm (Yang et al. 2005). To further analyze sequences for positive selection, the MEME (Murrell et al. 2012) and FUBAR (Murrell et al. 2013) tools were implemented on the Datamonkey server (http://www.datamonkey.org; accessed April 2020; Pond and Frost 2005; Delport et al. 2010; Weaver et al. 2018) using the same alignments (excluding pseudogenes) that were used for the PAML analysis. Default significance thresholds were used for both tools (*P*-value of 0.1 for MEME and posterior probability of 0.9 for FUBAR).

### Homology Modeling

We used I-TASSER server (https://zhanglab.ccmb.med.umich.edu/I-TASSER/; Roy et al. 2010) to generate 3D models of the *A.forsteri* TLR exodomains. As homology templates for AfoTLR1B and AfoTLR2B, we selected human TLR1/TLR2 (PDBID: 2z7x), for AfoTLR4 we used mouse TLR4 (PDBID: 5ijb), for AfoTLR5 zebrafish TLR5b (PDBID: 3v44) and for AfoTLR7 mouse TLR7 (PDBID: 5gmh). For further analysis, only I-TASSER models with the highest *C* values were used (in all cases this confidence score was *C* > 0.5, estimated RMSD max. 5.7 ± 3.6 Å). To visualize the location of positively selected sites and functional residues and to measure their molecular distances we used the PyMOL Molecular Graphics System (version 2.3.3, Schrödinger, LLC).

### Phasing of TLR15 Haplotypes

*TLR15* haplotypes were inferred for individual birds using Beagle (Browning and Browning 2007; Browning et al. 2018). Data were prepared in accordance with GATK Best Practice recommendations (DePristo et al. 2011; Van der Auwera et al. 2013). First, HaplotypeCaller in GATK was used to reassemble reads in putative indel sites and assign per-sample genotypes, as well as infer physical phasing of variants on contiguous reads (Poplin et al. 2018). Per-sample genotypes were aggregated using CombineGVCFs in GATK and then joint-genotyping was performed using GenotypeGVCFs. Variants were filtered using VariantFiltration with parameters of read depth ≥ 6 and base quality ≥ 30. Following phasing using Beagle, bcftools (Li et al. 2009) was used to extract the two haplotypes per individual for further analysis.

### Fungal Lysate Preparation

*Saccharomyces cerevisiae* lysates were prepared as previously described (Boyd et al. 2012). For *A.fumigatus*, lysates were prepared by streaking a fresh Sabouraud dextrose agar plate (with chloramphenicol) with a swab from the lung of a Humboldt penguin with aspergillosis at necropsy, growth at 37 °C for 48 h, then homogenization in a bead beater (Biospec) in ice-cold sterile phosphate-buffered saline (PBS), followed by centrifugation at 13,000 × g at 4 °C for 15 min to clear the lysate.

### Cloning of TLR15 Constructs

Gblocks comprising the full-length sequences of North fulmar (*F.glacialis*), Emperor penguin (*A.forsteri*), consensus *Eudyptes* intact *TLR15*, sequences were designed (IDT), and cloned into the p3×FLAG-CMV-14 vector (Sigma) using a Gibson assembly approach (Gibson et al. 2009). Chicken TLR15 in pTarget (Promega) was used previously by (Boyd et al. 2012). Mouse CD4-TIR15 and *Eudyptes*-chicken TIR15 chimaeras were designed as previously described (Medzhitov et al. 1997; Boyd et al. 2012) and cloned into pTarget (CD4 chimaeras) or p3×FLAG-CMV-14 (chicken chimaera).

### Transient Transfection and Stimulation

All constructs were transiently transfected into HEK-Blue Null1 cells (which express the SEAP reporter gene under the control of the IFN-β minimal promoter fused to five NF-κB and AP-1 binding sites; Invivogen) using TransIT-2020 (Mirus) in 24-well plates with three technical replicates per condition. Cells were maintained in high glucose Dulbecco’s Modified Eagle’s Medium (Gibco). Forty-eight hours after transfection, cell culture medium was replaced with medium containing the appropriate agonist (*A.fumigatus* or *S.cerevisiae* lysate at 1 µg/ml, or medium control). After a further 24 h, cell supernatants were aspirated to measure the enzymatic activity of the secreted alkaline phosphatase (SEAP), which is a proxy for NF-κB activation. Supernatants were mixed with p-Nitrophenyl Phosphate (Sigma) according to the manufacturer’s instructions, and absorbance was measured at 405 nm on a FLUOstar Omega microplate reader (BMG Labtech) at 37 °C. Differences between means were tested statistically using an analysis of variance model with Dunnett’s test (Dunnett 1955) for multiple comparisons to a control in R (v. 4.0.2).

### Immunoblotting

Immunoblotting was performed on the same samples as the SEAP assay to confirm protein expression. Briefly, cells were lysed directly in NuPAGE LDS Sample Buffer (4X; Novex), supplemented with 0.1 M DTT, boiled for 10 min at 95 °C, resolved on a 4–20% gradient gel (Bio-Rad), transferred using a semidry system (Bio-Rad) at constant current of 200 mA for 1 h, blocked using StartingBlock PBS blocking buffer (Thermo Scientific), probed using mouse monoclonal anti-FLAG (M2; 1:1,000; Sigma) overnight at 4 °C, then after washing, probed with a goat anti-mouse HRP-conjugated secondary antibody (Cusabio; 1:10,000), and visualized on an ImageQuant LAS 4000 (Cytiva) using SuperSignal West Pico PLUS chemiluminescent substrate (Thermo Scientific). 

## Supplementary Material

Supplementary data are available at *Molecular Biology and Evolution* online.

## Supplementary Material

msab354_Supplementary_Data

## Data Availability

All sequence data analyzed in this study are available from GenBank using accessions: MW793727—MW793735 (*TLR1A*); MW793736—MW793744 (*TLR1B*); MW793745—MW793756 (*TLR2A*); MW793757—MW793767 (*TLR2B*); MW793768—MW793786 (*TLR3*); MW793787—MW793805 (*TLR4*); MW793806—MW793824 (*TLR5*); MW793825—MW793843 (*TLR7*); MW793844—MW793854 (*TLR15*); MW793855—MW793869 (*TLR21*). Population-level *TLR15* data are available with accessions: MW805883–MW806032.
