# Supplementary material for: Adaptation and Cryptic Pseudogenization in Penguin Toll-Like Receptors
Source: Mol Biol Evol. 2021 Dec 13;39(1):msab354. doi: 10.1093/molbev/msab354 (PMC8788240; doi:10.1093/molbev/msab354)
Supplement: msab354_Supplementary_Data [file msab354_supplementary_data.pdf]

Supplementary information for:

Fiddaman, et al. (2021) Adaptation and cryptic pseudogenization in penguin Toll-like receptors

TLR1B

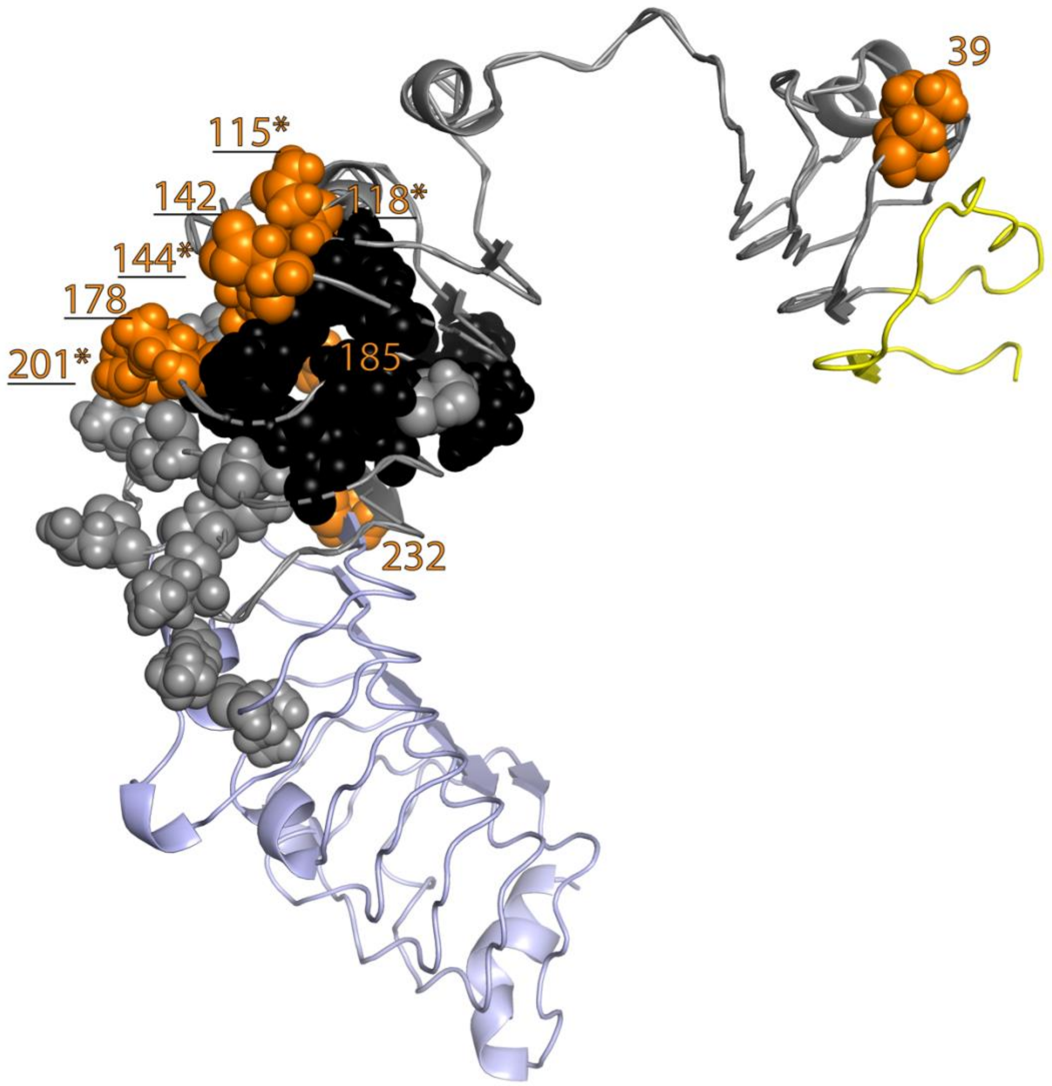

Supplementary Figure S1. Homology models of TLRs showing positions of positively selected sites. Legend is the same as Figure 4 of the main manuscript.

TLR2B

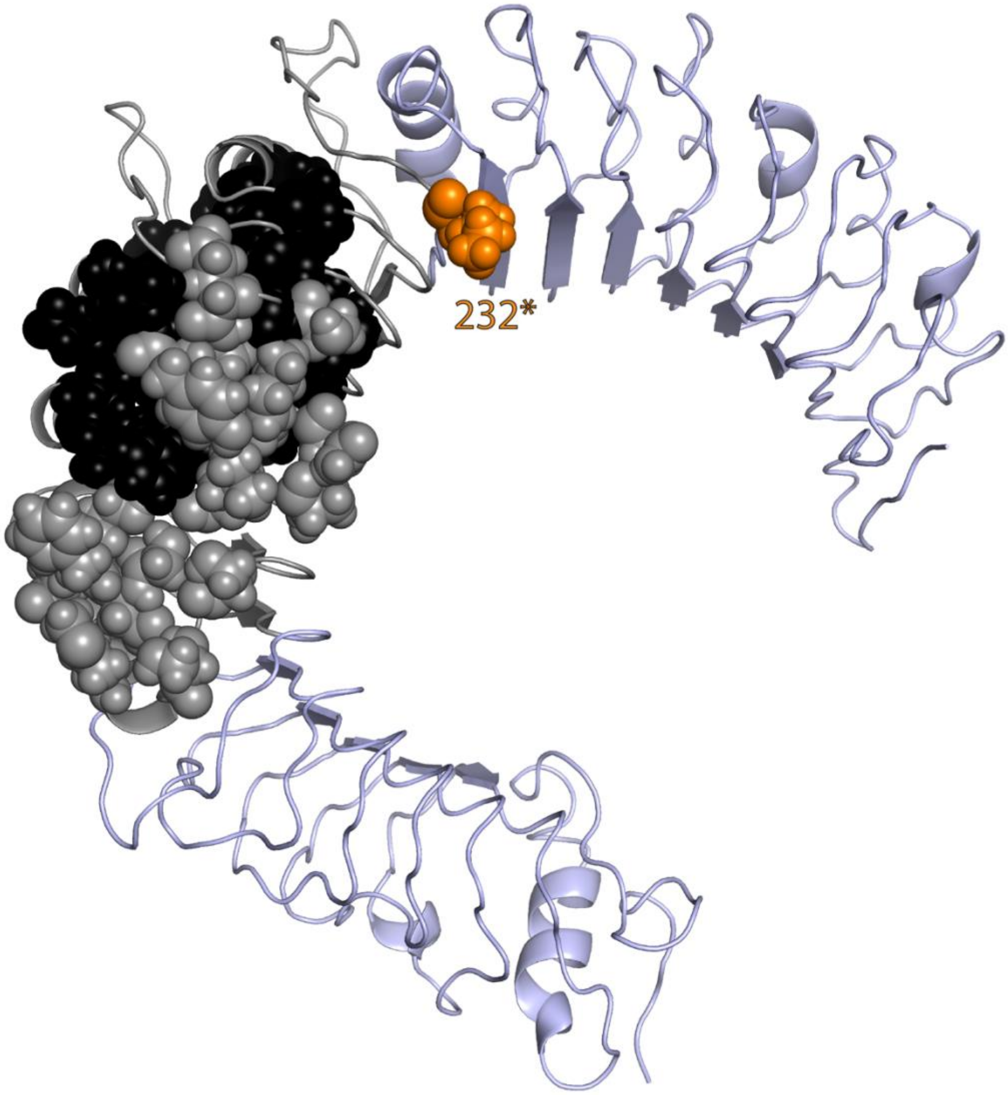

Supplementary Figure S1. Homology models of TLRs showing positions of positively selected sites. Legend is the same as Figure 4 of the main manuscript.

TLR4

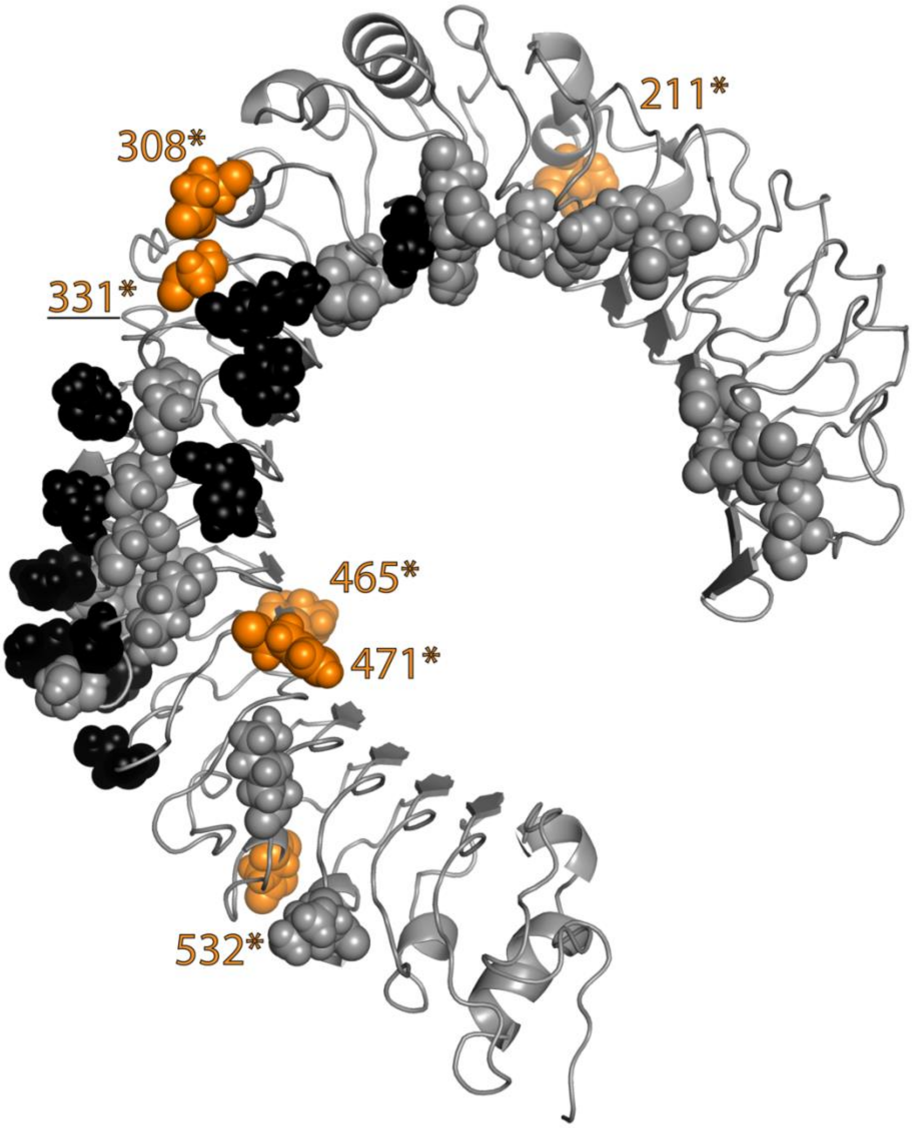

Supplementary Figure S1. Homology models of TLRs showing positions of positively selected sites. Legend is the same as Figure 4 of the main manuscript.

TLR5

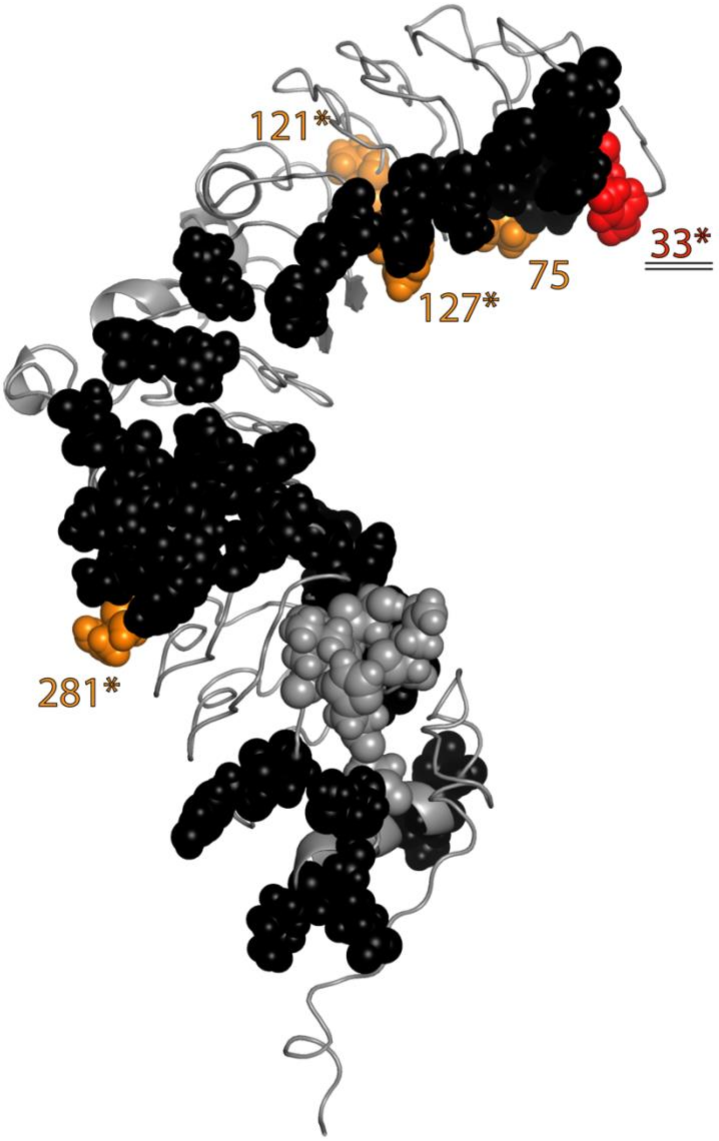

Supplementary Figure S1. Homology models of TLRs showing positions of positively selected sites. Legend is the same as Figure 4 of the main manuscript.

TLR7

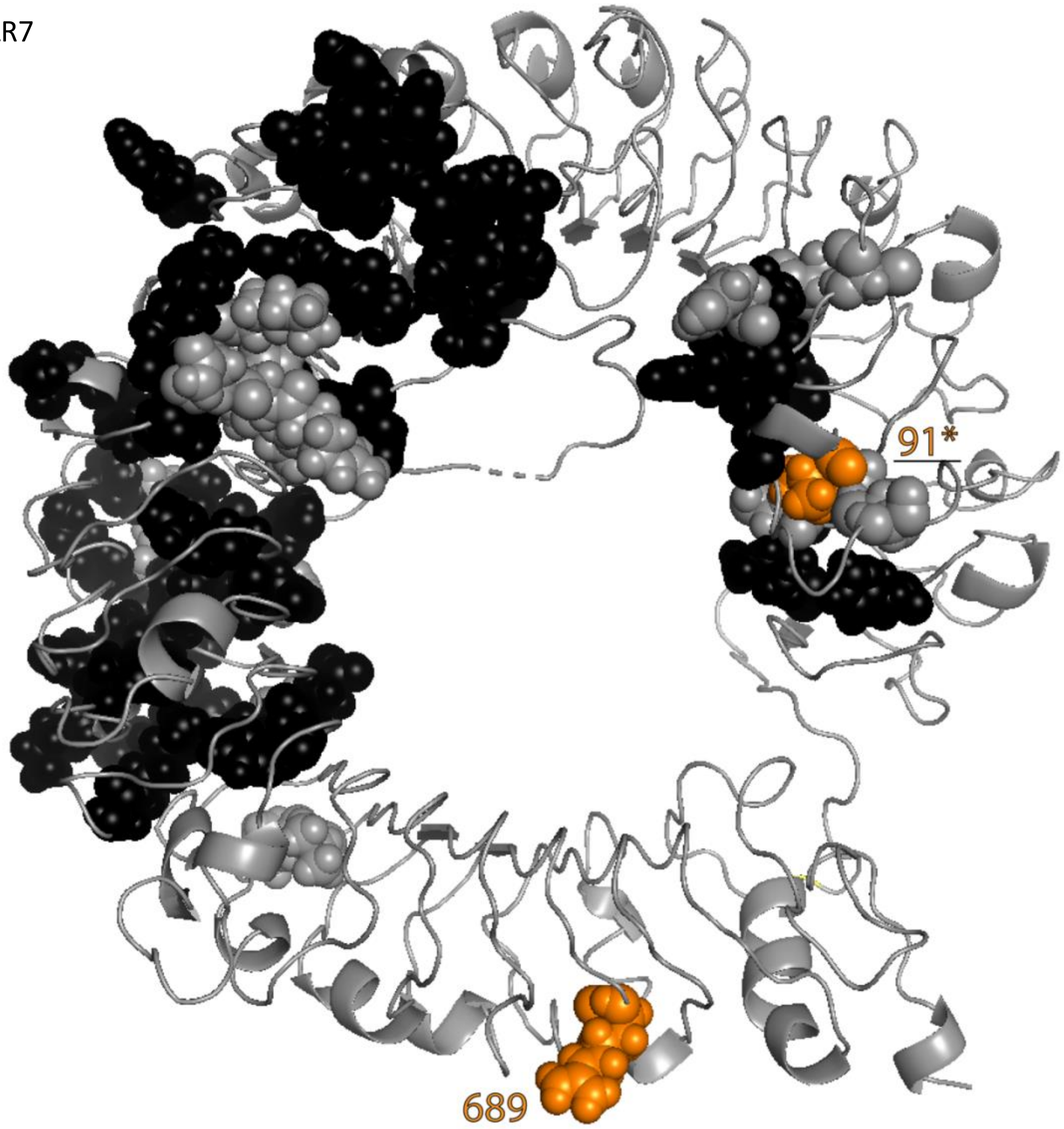

Supplementary Figure S1. Homology models of TLRs showing positions of positively selected sites. Legend is the same as Figure 4 of the main manuscript.

Supplementary Information S2: Coverage depth of TLR15 loci relative to all known coding sequences

Background
One explanation for the pseudogenization of TLR15 is that the gene underwent duplication, and one copy experienced functional redundancy and degradation in the genome. Even though this scenario does not fit well with TLR15 (there were several homozygous pseudogene haplotypes and we did not observe any triallelic sites at the locus), another method of detecting duplication is through analysis of read coverage of the locus.

Methods
BAM files from analysis conducted in Pan, et al. (2019) were obtained for three penguin species (one non-*Eudyptes* spp. and two *Eudyptes* spp.: *Spheniscus humboldti*, *Eudyptes robustus*, *Eudyptes chrysocome*. Using coordinates derived from known coding sequences from Pan, et al. (2019), average depth of coverage was calculated for each region using samtools depth and awk. Using BLAST, the genomic position of the TLR15 locus was determined for each species and average coverage was determined for this region. Finally, average depth of coverage was plotted in a pairwise fashion between pairs of the three genomes.

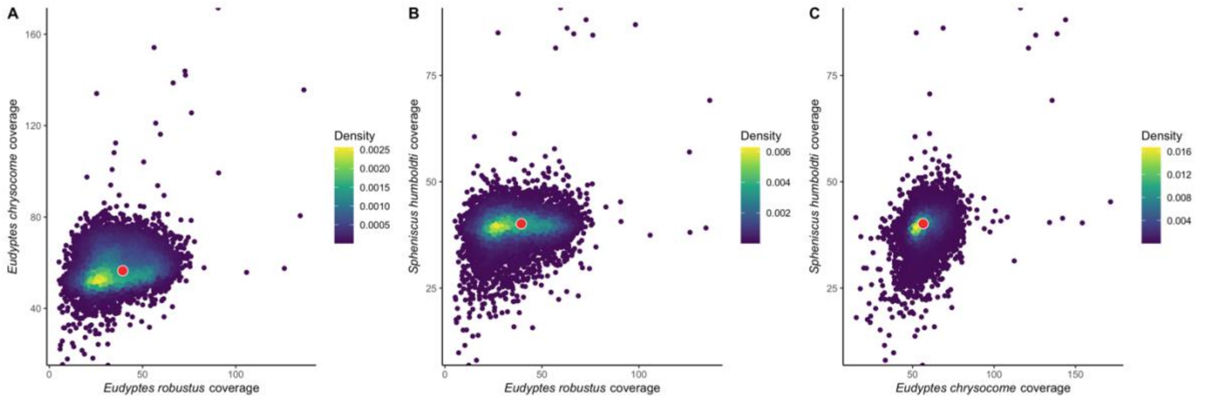

Figure S2: Pairwise depth of coverage of TLR15 and all other known coding sequences in the genomes of three penguin species. Depth of coverage for all coding loci are plotted (small points) and coloured according to their density. Depth of coverage for TLR15 is overlaid (red point).

Results
In each pairwise comparison, depth of coverage for TLR15 lies well within the high-density cluster of coding loci in the genome.

Interpretation
Since depth of coverage for TLR15 is comparable to the vast majority of coding sequences in the genome, it is highly likely that TLR15 has not undergone duplication in the *Eudyptes* lineage. This, along with the presence of homozygous mutations lack of triallelic sites, implies that TLR15 is a unitary pseudogene in the *Eudyptes* penguins.

References
Pan HL, Cole TL, Bi XP, Fang MQ, Zhou CR, Yang ZT, Ksepka DT, Hart T, Bouzat JL, Argilla LS, et al. 2019. High-coverage genomes to elucidate the evolution of penguins. Gigascience 8.

TLR	Model	lnL	2(ΔlnL)	p-value
1A	M1a	-1821.206256	1.00E-06	1
1A	M2a	-1821.206255		
1A	M7	-1821.207512	0.00024	1
1A	M8	-1821.207272		
1B	M1a	-1335.556606	13.99	0.000918
1B	M2a	-1328.562989		
1B	M7	-1335.556601	13.99	0.000918
1B	M8	-1328.563326		
2A	M1a	-1063.044738	1.58	0.45
2A	M2a	-1062.25387		
2A	M7	-1063.061381	1.62	0.45
2A	M8	-1062.25387		
2B	M1a	-936.166981	3.97	0.14
2B	M2a	-934.166981		
2B	M7	-936.386588	4.41	0.11
2B	M8	-934.182354		
3	M1a	-4726.812656	4.043078	0.133
3	M2a	-4724.791117		
3	M7	-4726.81527	4.047772	0.132
3	M8	-4724.791384		
4	M1a	-4532.656932	14.015116	0.00091
4	M2a	-4525.649374		
4	M7	-4532.794629	14.29414	0.00079
4	M8	-4525.647559		
5	M1a	-4730.087159	24.504362	4.78E-06
5	M2a	-4717.834978		
5	M7	-4731.145327	26.619834	1.66E-06
5	M8	-4717.83541		
7	M1a	-5183.621165	7.169838	0.0277
7	M2a	-5180.036246		
7	M7	-5183.62674	7.16707	0.0278
7	M8	-5180.043205		
15	M1a	-4816.320022	7.523166	0.0233
15	M2a	-4812.558439		
15	M7	-4816.347303	7.545864	0.023
15	M8	-4812.574371		
21	M1a	-4313.32987	0.870012	0.647
21	M2a	-4312.894864		
21	M7	-4313.570862	1.346234	0.51
21	M8	-4312.897745		

Supplementary Table S1. Results from site models comparisons (M2/M2a and M7/M8) in the *codeml* program in PAML. Likelihood ratio tests were performed by calculating double the difference in log likelihood values between the alternative model (M2a or M8) and the null

model (M2 or M7). P values were obtained from the chi-squared distribution with 2 degrees of freedom (calculated in each case as the difference between the numbers of parameters included in each model). Comparisons with P values < 0.05 were considered to be statistically significant, and are highlighted in green. Note that only the non-gene-converted regions of the TLR1/2 family were included in the analysis.

TLR	Site	AA	Pos. prob.	Omega	SE	Domain	Chicken site	Chicken AA	Other study (ref)	Function	Function
TLR1B	39	P	0.958	6.353	2.271	ECD	43	L			
TLR1B	115	N	0.923	6.117	2.452	ECD	119	K	1	<5Å lipopeptide binding	11
TLR1B	118	T	0.927	6.15	2.429	ECD	122	I	1	<5Å lipopeptide binding	11
TLR1B	142	D	0.946	6.276	2.335	ECD	146	N	2	<5Å lipopeptide binding	11
TLR1B	144	K	0.999	6.592	2.004	ECD	148	V	1, 3	<5Å lipopeptide binding	11
TLR1B	178	I	0.926	6.141	2.435	ECD	182	I		<5Å lipopeptide binding	11
TLR1B	185	A	0.94	6.237	2.366	ECD	189	A			
TLR1B	201	L	0.954	6.326	2.295	ECD	205	S	3, 4	<5Å dimerization site	
TLR1B	232	T	0.935	6.2	2.394	ECD	237	F			
TLR2B	232	K	0.96	5.333	2.808	ECD	230	K	1		
TLR4	15	L	0.916	4.631	1.482	ECD	9	P	1, 4	Not modelled	
TLR4	211	A	0.978	4.872	1.142	ECD	205	T	3		
TLR4	308	E	0.979	4.874	1.138	ECD	302	N	1, 3, 4, 5		
TLR4	331	E	0.922	4.656	1.453	ECD	325	E	3, 4, 6, 7, 8	<5Å LPS and MD-2 dimerization	12, 13
TLR4	465	V	0.901	4.57	1.546	ECD	459	I	3		
TLR4	471	H	0.907	4.595	1.52	ECD	465	Y			
TLR4	532	D	0.91	4.604	1.511	ECD	526	N	3, 6		
TLR4	661	V	0.905	4.585	1.531	TMD	655	G	1, 3	Not modelled	
TLR4	800	R	0.903	4.576	1.54	TIR	794	R		Not modelled	
TLR5	20	C	0.902	4.72	1.541	ECD	20	C	1	Not modelled	
TLR5	33	L	0.985	5.068	1.033	ECD	33	M	1	FLA binding site (60)	14
TLR5	75	M	0.986	5.074	1.023	ECD	75	L	1		
TLR5	121	Q	0.988	5.082	1.007	ECD	121	Q	1		
TLR5	127	R	0.981	5.055	1.059	ECD	127	R			
TLR5	281	D	0.977	5.037	1.092	ECD	281	T	1, 9, 10		
TLR5	429	H	0.918	4.789	1.465	ECD	429	H		Not modelled	
TLR5	521	R	0.987	5.079	1.012	ECD	521	Q		Not modelled	
TLR5	660	V	0.917	4.784	1.472	TMD	660	I		Not modelled	
TLR5	827	V	0.98	5.048	1.071	TIR	827	I		Not modelled	
TLR5	845	Q	0.93	4.84	1.403	TIR	845	Q	1	Not modelled	
TLR7	91	V	0.905	2.132	0.384	ECD	101	V		<5Å dimerization site	15

TLR7	689	C	0.901	2.127	0.391	ECD	699	L	3, 4	Not modelled
TLR15	619	V	0.936	2.79	1.169	ECD	620	I		Not modelled

Supplementary Table S2: Locations of positively selected sites in penguin TLRs. In the final three columns, an indication is given as to whether the site has been identified as positively selected in other studies,

References

1 Velova H, Gutowska-Ding MW, Burt DW, Vinkler M. 2018. Toll-Like Receptor Evolution in Birds: Gene Duplication, Pseudogenization, and Diversifying Selection. Molecular Biology and Evolution 35:2170-2184.

2 Huang, Y., Temperley, N.D., Ren, L., Smith, J., Li, N., Burt, D.W., 2011. Molecular evolution of the vertebrate TLR1 gene family - a complex history of gene duplication, gene conversion, positive selection and co-evolution. Bmc Evol. Biol. 11, 149. doi:10.1186/1471- 2148-11-149

3 Wlasiuk, G., Nachman, M.W., 2010. Adaptation and Constraint at Toll-Like Receptors in Primates. Mol. Biol. Evol. 27, 2172–2186. doi:10.1093/molbev/msq104

4 Areal, H., Abrantes, J., Esteves, P.J., 2011. Signatures of positive selection in Toll-like receptor (TLR) genes in mammals. Bmc Evol. Biol. 11. doi:10.1186/1471-2148-11-368

5 Fornuskova, A., Vinkler, M., Pages, M., Galan, M., Jousselin, E., Cerqueira, F., Morand, S., Charbonnel, N., Bryja, J., Cosson, J.-F., 2013. Contrasted evolutionary histories of two Toll-like receptors (Tlr4 and Tlr7) in wild rodents (MURINAE). BMC Evol. Biol. 13.

6 Vinkler, M., Bryjova, A., Albrecht, T., Bryja, J., 2009. Identification of the first Toll-like receptor gene in passerine birds: TLR4 orthologue in zebra finch (Taeniopygia guttata). Tissue Antigens 74, 32–41. doi:10.1111/j.1399-0039.2009.01273.x

7 Nakajima, T., Ohtani, H., Satta, Y., Uno, Y., Akari, H., Ishida, T., Kimura, A., 2008. Natural selection in the TLR-related genes in the course of primate evolution. Immunogenetics 60, 727–735. doi:10.1007/s00251-008-0332-0

8 Shen, T., Xu, S., Wang, X., Yu, W., Zhou, K., Yang, G., 2012. Adaptive evolution and functional constraint at TLR4 during the secondary aquatic adaptation and diversification of cetaceans. BMC Evol. Biol. 12. doi:10.1186/1471-2148-12-39

9 Grueber, C.E., Wallis, G.P., Jamieson, I.G., 2014. Episodic Positive Selection in the Evolution of Avian Toll-Like Receptor Innate Immunity Genes. Plos One 9, e89632. doi:10.1371/journal.pone.0089632

10 Vinkler, M., Bainova, H., Bryja, J., 2014. Protein evolution of Toll-like receptors 4, 5 and 7 within Galloanserae birds. Genet. Sel. Evol. 46. doi:10.1186/s12711-014-0072-6

11 Jin, M.S., Kim, S.E., Heo, J.Y., Lee, M.E., Kim, H.M., Paik, S.-G., Lee, H., Lee, J.-O., 2007. Crystal structure of the TLR1-TLR2

heterodimer induced by binding of a tri-acylated lipopeptide. CELL 130, 1071–1082. doi:10.1016/j.cell.2007.09.008

12 Paramo, T., Piggot, T.J., Bryant, C.E., Bond, P.J., 2013. The Structural Basis for Endotoxin-induced Allosteric Regulation of the Toll-like Receptor 4 (TLR4) Innate Immune Receptor. J. Biol. Chem. 288, 36215–36225. doi:10.1074/jbc.M113.501957

13 Ohto, U., Yamakawa, N., Akashi-Takamura, S., Miyake, K., Shimizu, T., 2012b. Structural Analyses of Human Toll-like Receptor 4 Polymorphisms D299G and T399I. J. Biol. Chem. 287, 40611–40617. doi:10.1074/jbc.M112.404608

15 Yoon, S., Kurnasov, O., Natarajan, V., Hong, M., Gudkov, A.V., Osterman, A.L., Wilson, I.A., 2012. Structural Basis of TLR5-Flagellin Recognition and Signaling. Science 335, 859–864. doi:10.1126/science.1215584

TLR	PAML selected site	MEME (P value)	FUBAR (posterior probability)
TLR1B	39	0.67	0.49
TLR1B	115	0.18	0.955
TLR1B	118	0.17	0.959
TLR1B	142	0.32	0.87
TLR1B	144	0.08	0.984
TLR1B	178	0.27	0.881
TLR1B	185	0.21	0.886
TLR1B	201	0.02	0.947
TLR1B	232	0.2	0.899
TLR2B	232	0.09	0.984
TLR4	15	0.19	0.928
TLR4	211	0.17	0.964
TLR4	308	0.17	0.967
TLR4	331	0.21	0.94
TLR4	465	0.15	0.935
TLR4	471	0.23	0.927
TLR4	532	0.22	0.925
TLR4	661	0.27	0.902
TLR4	800	0.28	0.907
TLR5	20	0.38	0.878
TLR5	33	0.07	0.986
TLR5	75	0.43	0.818
TLR5	121	0.05	0.99
TLR5	127	0.28	0.947
TLR5	281	0.32	0.924
TLR5	429	0.18	0.926
TLR5	521	0.1	0.979
TLR5	660	0.18	0.931
TLR5	827	0.19	0.964
TLR5	845	0.19	0.931
TLR7	91	0.08	0.979
TLR7	689	0.28	0.953
TLR15	619	0.03	0.986

Supplementary Table S3: Analysis of positively selected sites in penguin TLRs using different methods. Alignments used for PAML analysis were also used for MEME and FUBAR analysis and results of MEME and FUBAR analyses are given here with reference to sites that were found to be positively selected in PAML analysis. The significance threshold for MEME was taken to be the default $P \leq 0.1$ and the significance threshold for FUBAR was taken to be the default posterior probability > 0.9. Significant sites in MEME and FUBAR analysis are shaded green.

Sampling Location	Latitude	Longitude	Sampled individuals (*N*)								
			E. chrysolophus	*E. schlegeli*	*E. moseleyi*	*E. filholi*	*E. chrysocome*	*E. sclateri*	*E. robustus*	*E. pachyrhynchus*	
**Antipodes Islands**	-49.69	178.77						1			
**Amsterdam Island**	-37.83	77.55			5, 1						
**Barnevelt Islands**	-55.82	-66.80					6, 1				
**Bird Island, South Georgia**	-54.01	-38.04	5								
**Bouvet Island**	-54.42	3.36	7								
**Crozet Islands**	-46.31	50.89	7			7, 1					
**Elephant Island, South Shetland Islands**	-61.11	-55.14	7								
**Falkland/Malvinas Islands**	-51.77	-59.50					8				
**Harrison Cove, New Zealand**	-44.63	167.91								1	
**Kerguelen Islands**	-49.34	69.33	7			7					
**Macquarie Island**	-54.63	158.86		6, 1		7					
**Marion Island**	-46.91	37.74	7, 1			7					
**Nightingale Islands**	-37.42	-12.48			7						
**The Snares, New Zealand**	-48.03	166.60							1		
**Terhalten Island, Tierra del Fuego**	-55.45	-67.06					7				**TOTAL**
**TOTAL by species**			41	7	13	29	22	1	1	1	115

References:

Vianna JA, Fernandes FAN, Frugone MJ, Figueiró HV, Pertierra LR, Noll D, Bi K, Wang-Claypool CY, Lowther A, Parker P, et al. 2020. Genome-wide analyses reveal drivers of penguin diversification. Proc Natl

Pan, et al. 2019. High-coverage genomes to elucidate the evolution of penguins. GigaScience 8(9) giz117, https://doi.org/10.1093/gigascience/giz117

Supplementary Table S4. Locations of samples used in this study. Reference genomes from Pan, et al. (2019) were used for the positive selection analyses, while genomic data from Vianna, et al. (2020) and unpublished data were used for the TLR15 population-level analysis. Since these sources of data used different seqeuencing technologies and assembly pipelines, only data from Vianna et al. were used for the TLR15 analysis, rather than a combined dataset. All data used in the analysis are available and details of accession numbers can be found in the 'Data Availability' section of the Main Text.

Supplementary Material – Identification of putative loss-of-function mutations in *Eudyptes* TLR15.

Including Supplementary Tables S5-S7 and Supplementary Figure S3

Alignment of intact *Eudyptes* TLR15 haplotypes with other birds

TLR15 protein sequences were downloaded from Ensembl or NCBI (chicken, ENSGALP00000013260; northern fulmar, XP_009585200.1, misannotated as TLR2; emu, ENSDNVP00000009442; blue tit, ENSCCEP00000009931; collared flycatcher, ENSFALP00000015961; helmeted guineafowl, ENSNMEP00000012998). These were aligned to TLR15 sequences from other penguins (n=11) and intact *Eudyptes* haplotypes (n=45, including the consensus TLR15 used in functional analysis). Next, residues were identified which are well conserved among penguins and other birds, but that are distinct in *Eudyptes* TLR15. These sites are presented in **Table S5**.

Amino acid site (relative to alignment)	Amino acid site (relative to chicken*)	Ancestral amino acid (consensus)	Derived (*Eudyptes*) amino acid	Chicken nuc. site coordinates
56	55	T	M	3:3024305
68	67	E	G	3:3024269
161	160	L	P	3:3023990
246	239	I	V	3:3023754
569	560	R/P/N	H	3:3022790
674	665	I/G/V	V	3:3022476
679	670	V/A	A	3:3022448
683	674	L	S	3:3022289
736	727	K/E	G	3:3022173
787	778	Q	R	3:3022136

Table S5. Amino acid and nucleotide positions of positions which are conserved among penguins and other birds, but are distinct in the *Eudyptes*. Red text indicates that the position is highly conserved across the whole of vertebrates (see **Figure S3**). *The extra intron-spanning methionine at the start of chicken TLR15 was removed prior to alignment.

Next, the variant positions were plotted against the conservation scores of TLR15 across 77 vertebrate genomes (data obtained from UCSC; **Figure S3**). Amino acid sites 161, 736 and 787 are highly conserved across vertebrates, but are different in *Eudyptes*, which could be indicative of a change in function. The mechanism of TLR15 activation has not been elucidated, so it is unclear which of these polymorphisms is the loss-of-function mutation, or whether multiple mutations resulted in the phenotype.

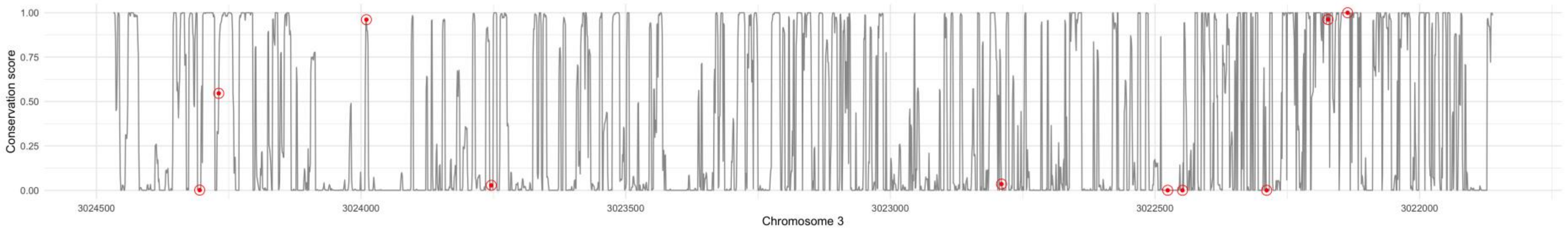

Figure S3. Per-nucleotide conservation scores for *TLR15* in chicken compared to 77 other vertebrate genomes (data obtained from UCSC). Sites that were identified as being distinct in *Eudyptes* compared to the rest of penguins are highlighted in red.

Next, variants found in the *Eudyptes* were used to replace equivalent residues in the chicken *TLR15* sequence, and the functional consequences predicted using Provean and SIFT. Provean analysis predicted L161P (chicken residue 160) and L683S (chicken residue 674) to be deleterious changes (**Table S6**), while SIFT also predicted L683S to be a deleterious change (**Table S7**). SIFT also predicted deleterious effects for T56M (chicken residue 55) and E68G (chicken residue 67), but since so few sequences were included as comparators for these sites, the prediction is of low confidence.

Variant	PROVEAN score	Prediction (cutoff= -2.5)
T55M	-1.630	Neutral
E67G	-1.484	Neutral
L160P	-2.555	Deleterious
I239V	-0.236	Neutral
R560H	-0.010	Neutral
I665V	-0.300	Neutral

V670A	-0.668	Neutral
L674S	-4.235	Deleterious
K727G	-1.862	Neutral
E727G	-1.749	Neutral
Q778R	0.337	Neutral

Table S6. Provean predictions for changes in TLR15 protein function. The chicken TLR15 sequence was modified with the indicated changes (sites are equivalent to the sites described in Table S5 for the *Eudyptes* penguins).

Variant	Median seq. conservation	Sequences represented	Function affected score	Outcome	Notes
T55M	4.32	1	0.00	AFFECT PROTEIN FUNCTION	**LOW CONFIDENCE**
E67G	3.44	2	0.01	AFFECT PROTEIN FUNCTION	**LOW CONFIDENCE**
L160P	3.44	2	0.06	TOLERATED	
I239V	3.34	4	0.57	TOLERATED	
R560H	3.14	46	0.13	TOLERATED	
I665V	3.03	48	1.00	TOLERATED	
V670A	3.03	48	0.22	TOLERATED	
L674S	3.09	47	0.00	AFFECT PROTEIN FUNCTION	
K727G	3.03	48	0.07	TOLERATED	

Table S7. SIFT predictions for changes in TLR15 protein function. As above, the chicken TLR15 sequence was modified with the indicated changes. Sites where SIFT indicated low confidence in predictions are indicated in ‘Notes’.

In summary, analysis of nucleotide conservation scores indicates that three non-synonymous variants in the *Eudyptes* (amino acid sites 161, 736 and 787) are otherwise highly conserved across vertebrates. This could be evidence of putative change in function at these sites. Moreover, Provean analysis predicts that

L161P is a deleterious change, which is consistent with a loss-of-function mutation. Continued work to elucidate the mechanism of action of TLR15 could provide insight into which mutations are the cause of the loss-of-function of *Eudyptes* TLR15.

	Mutation								
	C143G	C185A	681+A	1391+?	1826+T	1996+T	2273+31bp	Overall*	Total (N) in sp.
chrysocome	0.048	0.333	0.024	0.048	0.524	0.000	0.024	0.714	42
chrysolophus	0.000	0.350	0.000	0.000	0.375	0.025	0.463	0.763	80
filholi	0.321	0.089	0.000	0.054	0.446	0.018	0.000	0.750	56
moseleyi	0.000	0.000	0.000	0.000	0.000	0.542	0.000	0.542	24
schlegeli	0.000	0.417	0.000	0.000	0.417	0.000	0.583	0.917	12

*proportion of pseudogenised haplotypes (any causal mutation)

Supplementary Table S8. Frequencies of each pseudogene mutation in population analysis.
